# Ionic Liquids Toxicity—Benefits and Threats

**DOI:** 10.3390/ijms21176267

**Published:** 2020-08-29

**Authors:** Jolanta Flieger, Michał Flieger

**Affiliations:** 1 Department of Analytical Chemistry, Medical University of Lublin, Chodźki 4a, 20-093 Lublin, Poland; 2 Medical University of Lublin, Faculty of Medicine, Aleje Racławickie 1, 20-059 Lublin, Poland; michalflieeeger@gmail.com

**Keywords:** ionic liquids, (eco)toxicity, QSAR/QSPR, medical applications, bioprocessing

## Abstract

Ionic liquids (ILs) are solvents with salt structures. Typically, they contain organic cations (ammonium, imidazolium, pyridinium, piperidinium or pyrrolidinium), and halogen, fluorinated or organic anions. While ILs are considered to be environmentally-friendly compounds, only a few reasons support this claim. This is because of high thermal stability, and negligible pressure at room temperature which makes them non-volatile, therefore preventing the release of ILs into the atmosphere. The expansion of the range of applications of ILs in many chemical industry fields has led to a growing threat of contamination of the aquatic and terrestrial environments by these compounds. As the possibility of the release of ILs into the environment s grow systematically, there is an increasing and urgent obligation to determine their toxic and antimicrobial influence on the environment. Many bioassays were carried out to evaluate the (eco)toxicity and biodegradability of ILs. Most of them have questioned their “green” features as ILs turned out to be toxic towards organisms from varied trophic levels. Therefore, there is a need for a new biodegradable, less toxic “greener” ILs. This review presents the potential risks to the environment linked to the application of ILs. These are the following: cytotoxicity evaluated by the use of human cells, toxicity manifesting in aqueous and terrestrial environments. The studies proving the relation between structures versus toxicity for ILs with special emphasis on directions suitable for designing safer ILs synthesized from renewable sources are also presented. The representants of a new generation of easily biodegradable ILs derivatives of amino acids, sugars, choline, and bicyclic monoterpene moiety are collected. Some benefits of using ILs in medicine, agriculture, and the bio-processing industry are also presented.

## 1. Introduction

Ionic liquids (ILs) have been known since 1914. They are a class of salts with low melting points that is achieved by the high bulkiness and asymmetry of the ions, thereby preventing the molecule packing from promoting crystallization [1]. Typically, they contain large-size nitrogen-containing cations such as ammonium, imidazolium, pyridinium, piperidinium, pyrrolidinium, or a phosphorus-containing cation (eg, phosphonium) combined with an anion of weak coordination properties, for instance, halogen, fluorinated or organic anions.

The first generation of ILs, which appeared at the beginning of the 1960s, was interesting considering their unique, and tunable physical properties (e.g., viscosity, density, low volatility, conductivity, thermal stability, large liquid ranges, or hydrophobicity). However, this generation possessed drawbacks, because, among others, they were sensitive to the presence of water and air. As a result, in the 1990s, the second generation of ILs was created as an answer to the increasing need for solvents with tunable physical and chemical properties. Such task-specific ILs were prepared from independently modified cations and anions, enabling the design of new functional materials. The second-generation ILs were able to ensure the improvement of chemical reactivity, chiral recognition, solvatation, oxygen balance, UV blocking, high energy density, and many others. Furthermore, this class of ILs was more stable and environmentally benign as they were based on the biodegradable source of ions [2]. This group of ILs was the subject of countless studies reporting their numerous applications.

The beginning of the 21st century marks the appearance of the third generation of ionic liquids connecting desired biological properties with chosen physicochemical properties. This group contains examples of ILs which are used in medicine, for instance, as active pharmaceutical precursors or ingredients (APIs). Cations of known low toxicity and beneficial activity (antibacterial, antifungal, anticholinergic, local anesthetic) and anions of certain properties (emollients, vitamins, antibiotics, NSAID) have been used to obtain a set of the required biological properties of the ILs [3,4,5].

Currently, the group of compounds referred to as ionic liquids is divided into different subgroups. An interesting group of ILs is the switchable IL group (SILs) known also as reversible ILs (RILs). Components of this group may change their state from a neutral to ionic by exposure to external activating factors such as the capture of carbon dioxide, sulfur dioxide, extraction of oil from soybeans, extraction of hemicellulose from pines and oil refining [6].

Traditionally, ILs are defined as salts with melting points below 100 °C. If the melting point is located between 25 °C and 100 °C, ILs are in the solid state, that is why they referred to as “frozen” ILs. Several years ago, the ILs chemistry has been utilized to tune the properties of solid-phase organic salts with melting point range of 25–250 °C [7]. This new group of chemical compounds was defined as a group of uniform materials based on organic salts (GUMBOS) [8,9,10,11,12]. GUMBOS can be categorized as solid-phase ionic liquid (IL). Utilizing IL chemistry, solid-phase materials properties such as, among others, magnetism, melting point, hydrophobicity, fluorescence quantum yields, nanoformulations, aggregation, viscosity, and even cytotoxicity are tunable, and can be tailored to a specific task. The GUMBOS, or nanoGUMBOS can be obtained by varied methods such as a reprecipitation [13,14], hard templating and the melt–emulsion–quench method [7]. Warner et al. proved that the fluorescence, magnetism and chirality of the parent material could be retained owing to addition of very low concentrations of nanoGUMBOS [15]. In 2012, Warner et al. [11] synthesized a new GUMBOS compound being a phosphonium derivative with a dysprosium ion, possessing toxicity toward cancer cells. A dysprosium ion as a fluorescent as well as paramagnetic component enables one to classify this product to a special class of ILs known as magnetic ionic liquids (MILs). Owing to their paramagnetic susceptibilities at room temperature, the molecular motion of MILs can be controlled by an external magnetic field. Hayashi et al. examined the paramagnetic properties of 1-butyl-3-methylimidazolium tetrachloroferrate(III) [BMIM][FeCl_4_] for the first time [16]. Till now different MILs containing transition and lanthanide metal-based anions including [MnCl_4_^2−^], [CoCl_4_^2−^], [GdCl_4_^1−^] have been synthetized [17]. MILs have found application in extraction techniques owing to the possibility of incorporating specific functional groups that can enhance the extraction, and because of their paramagnetic properties, enabling separation of MILs using an external magnetic field and finally resistance to agglomeration. We can distinguish three classes of iron-based MILs with different cations, namely monocationic, dicationic, and tricationic MILs. The highest effective magnetic moment (11.76 Bohr magnetons (µB)) was reported for tricationic imidazolium-based MILs comprising three [FeCl_3_Br^−^] anions. Owing to their high hydrophobicity and relatively low viscosities, ammonium, phosphonium and a few dicationic imidazolium-based MILs have been used as extraction solvents for the preconcentration of nucleic acids and polyaromatic hydrocarbons from complex sample matrices [18,19]. Another class of MILs is represented by water-soluble N-substituted imidazole ligands (with butyl-, benzyl-, or octyl-groups as substituents) coordinated to different metal centers (Ni^2+^, Mn^2+^, or Co^2+^) as cations. These MILs appeared to be useful for in situ dispersive liquid–liquid microextraction (DLLME) of long and short double-stranded DNA. During the extraction, MILs react with the bis[(trifluoromethyl)sulfonyl]imide anion undergoing precipitation together with DNA, ensuring extraction efficiencies ranging from 42 to 99% [20].

Solid-state ILs can be produced also by polymerization of an ionic liquid (IL) monomer [21]. Polymeric ionic liquids (PILs) offering a broad range of physicochemical properties have emerged recently as an interdisciplinary topic across multiple research fields covering materials science. For example, has served as a common even though. Some studies have reported the complicated and time-consuming PILs synthesis process [22,23] as well as the development of ionic nanoparticle networks [24]. Other subgroup of ILs is constituted by ILs with long carbon chain substituents within the cationic headgroup. These ILs are able to form micellar aggregates in aqueous environment above a certain concentration known as the critical micelle concentration (CMC). Taking into account, classical surfactants classification, into cationic, anionic, nonionic, and zwitterionic, ILs, possessing above ability, exhibit characteristics of cationic surfactants. Till now, only a few tens of IL-based surfactants covering monocationic and dicationic derivatives of imidazolium, the pyridinium, the pyrrolidinium cations connected by an alkyl linkage chain of varying lengths have been reported [25].

Because of some threats connected with the spread of ionic liquids in the environment, deep eutectic solvents (DES) were recognized as potential alternatives of ILs used as liquids or lubricants [26]. It should be emphasized that some of the characteristics of ILs are shared by DESs. One of them is an extremely low volatility, and tunable properties which can be adjusted by properly selecting the nature of the hydrogen bonding pairs to display a wide liquid range, water-compatibility, non-flammability, non-toxicity, biocompatibility or biodegradability. DESs represent mixtures consisting of at least one hydrogen bond donor (HBD) and one hydrogen bond acceptor (HBA). The resulting liquid mixture shows an unusual decrease of the melting temperature in comparison to the melting temperature of the pure components, caused by entropy of mixing, hydrogen bonding and van der Waals interactions [27,28]. At the beginning, hydrophilic DESs were synthesized from amides and choline chloride [29]. A few years ago hydrophobic DESs were reported and applied as extracting solvents [30,31]. Nowadays, the trend of using more natural components of DESs such as terpenes can also be observed. However, there is still the lack of detailed investigations on their toxicity [32,33,34].

Nowadays, ILs have found widespread applications due to their superior and unique solvent properties compared with conventional organic solvents. ILs as viscous liquids present a great capacity to dissolve numerous inorganic and organic substances, for chemical reactions or separation processes, as electrolytes in batteries, and photovoltaic devices, as a medium for electrodeposition of metals, and many others. Numerous reviews have collected data concerning the areas where they could be applied [35,36,37,38,39].

Much of the published data considers the evaluation of the physicochemical properties of ILs covering decomposition temperatures, ionic conductivities, viscosities, mutual solubilities with water, densities, melting point or surface tension. Such a wide range of properties is closely related to the structure of ILs, i.e., the involvement of both the anion and cation. Therefore, ILs can be tailored to have the required properties by changing the cation or the anion using an appropriate molecular design strategy [40,41].

Fewer studies, however, take into account their toxicities. For a long time, it was believed that these chemicals were safe for the environment because they are composed of charged units, they exhibit high thermal stability and low vapor pressure at normal temperatures and are considered non-volatile, therefore, they do not lead to evaporation into the atmosphere that can result in smog formation, ozone depletion, and climate change [42]. Furthermore, the volatility of solvents exposes humans and animals to inhalation, or the possibility of explosions considering their lower flashpoints. From this point of view, ILs still can be considered as less harmful compounds, although, in 2006, Smiglad reported several combustible ILs [43]. Thus, diffusion into the atmosphere is negligible, but their water solubility can still allow ILs to enter into the aquatic compartment easily, which increases a potential threat to the aquatic environment and organisms living there. Furthermore, ILs can persist in this environment for a long time because of their high stability making them poorly biodegradable [44].

This class of compounds, which was considered in the early days of their application, as non-noxious, nowadays has been undergoing evaluation according to (eco)toxicity at several biological levels with aim to estimate the risks to the environment. So far, some main trends concerning toxicological studies for ILs can be distinguished. These are the following: toxicity for humans covering cytotoxicity utilizing human cells such as Caco-2, HeLa, breast cancer cell, human colon carcinoma HT29, HepG2 (liver cancer), AGS (stomach adenoma), A549 (lungs cancer), inhibition of enzymes activity (acetylcholinesterase, AMP deaminase, alcohol dehydrogenase ect.), toxicity for patogenic bacteria (*Staphylococcus aureus, Enterococcus faecalis, Mycobacterium smegmatis, Pseudomonas aeruginosa, Klebsiella pneumonia, Candida albicans*); toxicity manifesting in aqueous ecosystem towards organisms such as bacteria (*Vibrio fischeri*), algae (*Selenastrum capricornutum, Oocystis submarina*), diatoms (*Cyclotella meneghiniana*), mollusks (*Physa acuta*), vertebrates (*Danio rerio, Rana nigromaculata, Cyprinus carpio*), plants (*Lemna minor*); impact on organisms habitating terrestrial environments such as bacteria (*Escheria coli*), invertebrates (*Eisenia fetida, Caenorhabditis elegans, Folsomia candida*), vertebrates (*Rattus norvegicus*), plants (*Triticum aestivum, Lepidium sativum, Capsicum annuum*); investigation of the relation between structure versus toxicity for ILs, and synthesis of a new generation of easily biodegradable ILs synthesized from renewable sources.

Numerous studies have shown that ILs exhibit different degrees of toxicity in species ranging from bacteria to higher organisms, and that the toxicity primarily depends on the cationic moiety and its side-chain length. Recently, there has also been evidence that anions can contribute to toxicity. It has been already demonstrated that most ILs based on imidazole exhibit higher toxicity in comparison to common organic solvents such as methanol and dichloromethane [45,46].

Taking into account the influence of ILs at the enzymatic level, the ability to inhibit many enzymes has been observed. The effect of inhibiting acetylcholinesterase, resulting in distortion of many neurological processes seems to be particularly dangerous. It has been proven that the pyridinium and imidazolium-based ILs are responsible for the inhibition of the activity of enzymes [47,48].

Toxic effects of ILs on either microorganisms [49,50,51] or mammalian cells [52,53] have been observed. Nowadays, there is no doubt that ILs exhibit toxicity and may pose hazards to human health and the natural environment. Initially, the studies of the ecotoxicity of ILs concerned the aquatic environment. This was due to the very good solubility of most ILs in water. A standard ecotoxicological bioassay in Europe it is DIN EN ISO 11,348 procedure. Moreover, there are several official standards of particular countries such as ASTM method D5660-1995 in USA, DIN38412-1990 in Germany, Germany, AFNOR T90-320-1991 in France, or ISO11348-3-1998 in Spain. The bioluminescence inhibition measurements of different microorganisms or enzymes like the *Vibrio fischeri* (*Photobacterium phosphoreum*) utilized as the test for acute toxicity.

In 1979, the global standard for the toxicity of water and solid samples, known as Microtox (ISO 11348-3, 1998), was presented in the USA. It is a rapid, cost-effective, and accurate test, compliant with ISO, ASTM, EPA, DIN, NNI, AFNOR, SNV, and other standards, for the assessment of acute toxicity and mutagenicity. Microtox is available for salt and brackish waters (MICROTOX marine) and for soil and sediments (MICROTOX solid phase). The tests enable determination of the mean EC_50_ value. In order to to enlarge the knowledge about the hazard potentials of ILs to the environment, the investigation is carried out using the flexible (eco)toxicological test battery covering different trophic levels from the molecular up to the organismic level. This test battery evaluates such parameters as growth inhibition of wheat (*Triticum aestvum*), cress (*Lepidium sativum*), duckweed (*Lemna minor*), reproduction inhibition of a soil invertebrate (*Folsomia cadida*) and limnic unicellular green algae (*Scenedesmus vacuolatus*), inhibition of enzyme activity (acetylcholinesterasae), viability of mammalian cells (IPC-81), luminescence inhibition of marine bacterium (*Vibrio fisheri*) [50,54].

Classification of ILs in terms of their toxicity can be made on the basis of measured parameters, such as the EC_50_. The EC_50_ value represents effective concentration resulting in 50% reduction of growth or reproductive activity, of the exposed organisms relative to the control. The EC_50_ range from 100 to 1000 mg of L^−1^ corresponds to no toxicity, the range of 10–100 mg L^−1^ indicates moderate toxicity, 1–10 mg L^−1^ corresponds to mild toxicity, 0.1–1 mg L^−1^ relates to high toxicity, 0.01–0.1 mgL^−1^ is for extremely toxic, and EC_50_ less than 0.01 mgL^−1^ marks super toxic xenobiotics [55]. In 2003, Jastorff et al. performed multidimensional risk analysis utilizing different ecotoxicological indicators such as: release, spatiotemporal range, bioaccumulation, biological activity to predict the risk ILs for the environment. However, the uncertainty of the results pointed out that more data concerning degradation, biotic/abiotic transformation, and toxicity are required to classify ILs as harmful compounds [56].

Other indices frequently applied for estimation of IL toxicity [54,57] are the following: LC_50_—lethal concentration/dose that kills half the members of a population tested in a specified time; IC_50_—inhibitory concentration resulting in 50% inhibition of the activity of biological or biochemical systems; LD_50_—median lethal dose; MIC—minimum inhibitory concentration, the lowest concentration that inhibits visible growth of a microorganism after overnight incubation.

Without doubts, most tests showed a negative effect of ILs on aquatic organisms. Therefore, most authors now question the term “green solvents”, which has been wrongfully assigned to ILs [58,59,60,61,62,63,64,65]. Despite the proven toxicity of ILs, a few companies have started their industrial use. For example, in 1998, the French Petroleum Institute included ILs to the production of polybutene, in 2003, *N*-alkylimidazole was approved as an extractant through BASF’s Biphasic Acid Scavenging Utilizing ILs (BASIL) process [66]. Therefore, studies providing information about the toxicity of ILs appear to be indispensable and even mandatory, prior to their widespread applications and potential release to the environment. This approach can prevent pollution and avoid future environmental clean-up costs. Moreover, the toxicity examination can help guide the molecular design of ILs with reduced hazard potential.

This review presents the current data devoted to the aquatic and territorial environmental and humans impacts of ILs, with special emphasis on directions of designing safer ILs. On the other hand, the benefits of using ILs in bioprocessing and medicine have been emphasized. There is some evidence for utilizing ILs with antibacterial, antifungal, and local anesthetic activity for medical purposes. Moreover, they can be applied for drug production and as components of delivery systems [3].

## 2. Toxicity against Microorganisms

Microorganisms are indispensable for the environment since they take part in the carbon and nitrogen cycles. In the literature, many reports concerning the toxicity of ILs to bacteria can be found. Numerous papers concern assessment of the toxicity of ILs to lactic acid-producing bacterium [49,67], *Vibro fischeri*, *Escherichia coli*, *Pichia pastoris*, *Bacillus cereus* and others [68] (Table 1).

The toxicity of ILs appeared to be more dangerous for bacteria in comparison to common solvents like phenol, toluene, benzene, and varies according to the species. Gouveia et al. [69] has suggested that toxicity against bacteria is not governed by the same rules established for higher organisms. The authors have found significant differences between the sensitivity of the soil bacteria, namely *E, coli* and *B. subtilis*, to imidazolium, pyridinium, and the choline-amino-acid-derived ILs. It appeared that ILs were more toxic to *B. subtilis* (Gram + bacteria), which was inhibited by [Cho][Gln] and [Cho][Met], than to *E. coli* (Gram—bacteria). Other research [69,70,71] has also confirmed that Gram-positive bacteria are more susceptible to ILs as compared to the Gram-negative due to different interactions of ILs with the peptidoglycan and lipid components of the cell wall.

Published reports have proven that in order to evaluate the toxicity against different organisms including bacteria, three structure components should be considered: the head group, the substituents in the head group, and the kind of anion [40,48,72,73,74]. It was found that the activity of bacteria decreased with increasing alkyl chain length on the imidazolium cations. However, some reports do not support this hypothesis. In 2014, Czekański et al. [72] tested a series of benzoazole ILs and evaluated their antimicrobial and antifungal activities. Among the synthesized ILs only a few of them, namely [BMIM][TBO], [HMIM][TBT] and [EMIM][TBI], exhibited inhibition zones against the Gram-positive *B. subtillis* and *P. aeruginosa.* It is interesting to note that regarding the bacteria tested, changing the head group in ILs structure or the length of the alkyl chain attached to the head group did not affect the antibacterial activity.

Cornmell, et al. [75] proved, using FT-IR spectra, that the ILs were accumulating within the cells of *Escherichia coli*. The FT-IR spectra were recorded against the background of the cell, demonstrating that the toxic ILs such as chlorides, [P_6,6,6,14_][Cl] and [N_1,8,8,8_][Cl] were accumulated more rapidly than the biocompatible water-immiscible ILs, trihexyltetradecylphosphonium bis(trifluoromethyl-sulfonyl)imide [P_6,6,6,14_][NTf_2_], and methyltrioctylammonium bis(trifluoromethylsulfonyl)imide [N_1,8,8,8_][NTf_2_]. It was seen that the [P_6,6,6,14_][NTf_2_] was trapped in the membrane and was not transferred into the cytoplasm.

### 2.1. Lactic Acid Bacteria

Lactic acid bacteria (LAB) fermentation has found numerous applications in the dairy industry, the meat industry, fermented vegetable production as well as production of probiotic ingredients in various products [79,80]. The major problem in the application of LAB culture as probiotics is the reduced growth of biomass due to inhibition action of lactic acid as an end-product which causes the decrease of the pH of the medium, and finally, disables the cellular functions of bacteria [81,82]. Therefore, to eliminate this negative effect of lactic acid during fermentation, it ought to be removed in situ from the culture. In 2003, Schiraldi et al. [80] suggested a dire need to develop efficient strategies enabling maintaining lactate concentration in the culture at below toxic level. In literature, we can find some examples of fed-batch fermentation as a method useful to overcome the end-product inhibition in LAB fermentation [82,83,84]. However, it appears that this methodology is often inefficient due to high osmotic pressure excided 2416 mOsm kg^−1^ and accumulation of acid anions and various metabolites [85].

The traditional approach used to overcome product suppression effect is by the addition of bases such as calcium hydroxide ammonium hydroxide, sodium hydroxide, calcium carbonate, trimethylamine, and dimethylamine to neutralize the acid formed and precipitate the insoluble salts. This process, however, consumes high amounts of reactants and also produces solid wastes requiring a costly utilization.

Another approach, overcoming the end-product inhibition effect, is solvent extraction fermentation [86,87], which is ineffective due to a high amount of toxic organic solvents needed and hydrophilic nature of lactic acid [88].

A more sophisticated approach offers multistep electrodialysis fermentation with an ion-exchange membrane [89,90]. However, this method has some disadvantages such as membrane fouling, high operating cost, and deionization of the culture broth [91].

Recently, we can observe a growing interest in aqueous two-phase systems (ATPS) in areas concerning biotechnology. This extracting system appeared to be useful and very efficient for the purposes of recovery and purification. The mechanism of ATPS is based on the partitioning process of target molecules between two liquid phases which are formed by mixing a polymer and a kosmotropic salt or eventually two polymers and water [92,93].

ATPS has also found its application for the removal of lactic acid. Dissing and Mattiesson, in 1994 [94], described ATPS containing a poly(ethyleneimine) (PEI) creating a lower phase accumulating lactate and hydroxyethylcellulose (HEC) at the top of the system extracting cells. Another two-phase system composed of ethylene oxide-propylene oxide/hydroxypropyl starch polymer-100 has been proposed by Planas et al. [95] with a purpose to remove lactate produced by *L. lactis* subsp. *lactis* 19435. They achieved an increase of lactate production from 27.8 mM in the first batch to 48.1 mM lactate produced in the fifth batch. In 2011, Aydogan et al. [96] proposed ATPS containing ethanol/dipotassium hydrogen phosphate. The obtained extraction yield for lactic acid achieved a level of 80%. Despite these promising results, the utility of the ATPS method for the industrial scale is limited due to the high cost of polymers.

To overcome the inhibition effect of lactic acid, extractive fermentation using adsorbents such as activated carbon, molecular sieves, polymeric adsorbent, ion exchange resins, zeolites can be applied [90,97,98,99,100]. Generally, this method allows removing target molecules and simultaneously maintaining of an actively growing culture in a culture medium at relatively low costs. Nevertheless, the method possesses some unwanted effects and limitations. Particular attention should be paid to adsorbents affinity toward nutrients in the fermentation medium [101], adsorbent capacity, and selectivity for lactic acid, and its biocompatibility with microorganisms. Another important requirement lowering the costs is the renewability, ensuring the re-use of the adsorbents.

### 2.2. Usefulness of Ionic Liquids in Bioprocessing

Lactic acid bacteria are microorganisms possessing fermentative ability. Nowadays, they are important mostly owing to their probiotic benefits as well as lactic acid production for different purposes. That is why; there is a dire need to invent more efficient methods for lactic acid removal from the culture in the integrated process of fermentation and separation. The main expectations towards the new method cover to maintain high cell concentrations and high recovery of lactic acid ensuring the lowest cost. The most critical problem appearing in operating bioproduction processes involving bacterial cells is the inherent toxicity of extracting solvents towards living organisms.

A few studies show the usefulness of ILs to improve the selectivity and yield in enzymatic reactions. The use of ILs as an alternative to conventional organic solvents has been already extensively recognized. The suitability of ILs has been also reported in the field of bioprocessing due to the improvement of the extraction selectivity and efficiency in enzymatic reactions [102,103,104]. A typical example of a bioproduction is lactic acid production by the fermentation process. Separation and purification steps developed for recovery of the raw material account for most of the production costs [105]. So far, solvent extraction or precipitation was used for an effective recovery of lactic acid from the broth [106,107]. However, the application of volatile organic solvents was connected with the inherent toxicity to bacterial cells as living organisms [108], and was also undesirable for environmental reasons.

Matsumoto et al. in 2004, examined the usefulness of imidazolium-based ILs as solvents in the extractive fermentation of lactate [67,78] by the use of *Lactobacillus rhamnosus* (NBRC 3863) as a lactic acid-producing bacterium. The authors found that extractions of organic acids such as acetic, glycolic, propionic, lactic, butyric and pyruvic by imidazolium-based hexafluorophosphates ([BMIM][PF_6_], [HMIM][PF_6_] and [OMIM][PF_6_]), without any extractant, is rather low. To achieve the extraction yield similar to conventional organic solvents (hexane, toluene), tri-*n*-butyl phosphate [TBP] should be added to IL as an extracting agent. The authors examined whether imidazolium-based ILs were toxic towards lactic acid-producing bacterium. The authors expressed the toxicity of ILs to *Lactobacillus rhamnosus* based on CFU values which are a measure of the survival rate of microbes. The value expresses the activity of microorganisms per amount of glucose consumed. It was confirmed that *L. rhamnosus* consumed glucose and produced lactate in the presence of the investigated imidazolium-based ILs. However, the activities of the bacteria generally were comparable to hexane and alkyl length in the imidazolium cation has almost no influence on CFU values.

## 3. Marine Toxicity of Ionic Liquids

The legend of ILs as “green solvents” comes from their negligible vapor pressure. However, the increasing applications of these salts in the broad range of industries requires justification for their safe use as a part of environmentally-harmless “clean technology”. There exists a great danger that ILs will become components of industrial wastewater and will be released into the environment. The long-term consequences and effects of such pollutants on biological processes in the aquatic environment are basically unknown. The first predictions concerning ecotoxicity of ILs appeared at the beginning of the XXI^st^ century. Theoretical simulations based on structure-activity relationships have been confirmed by experimental evaluations [50,52,56,109,110]. It is worrying that there exists a considerable structural similarity between certain cations of ILs and biologically active plant growth regulators or cationic surfactants which are known to have negative environmental effects.

One of them is chlormequat chloride (2-chloroethyltrimethylammonium chloride-CCC), described as a plant growth regulator by Tolbert in 1960 [111]. Despite its toxicity and side effects, it is commonly used to prevent lodging and to increase yields in wheat, rye, oats, and triticale [112]. Pernak et al. used CCC as substrate in the synthesis of the third generation ILs possible to use as herbicides and plant growth regulators. The authors synthesized new ILs namely, 2-chloroethyl-trimethylammonium and trimethylvinylammonium (2,4-dichlorophenoxy)acetates by combining CCC with (2,4-Dichlorophenoxy)acetic acid (2,4-D) which is a systemic herbicide as well as a synthetic auxin often used in laboratories for plant research [113].

In 2002, Gathergood and Scammells [114] discovered that imidazolium-based ILs only undergo a very small level of biodegradation. This was confirmed by Stepnowski in 2005 [115] indicating that imidazolium ILs are resistant to photodegradation. Thus, ILs may affect the aquatic environment by staying there for a long time because of the poor biodegradability. The threat that ILs can pose to aquatic ecosystems prompted the United States National Toxicology Program (NTP) in 2004 to conduct extensive toxicological studies of pyridinium, imidazolium, and pyrrolidinium ILs. The systematic toxicity studies have been documented in numerous works (Table 2).

ILs can affect aquatic ecosystems through the mortality of organisms, alteration of the demographic rates, changes of species interactions, alteration of biogeochemical processes and bioaccumulation occurring at different trophic levels. An assessment of the environmental hazards should contain the identification of the consequences observed as a result of interactions between chemical and different organisms in the environment and the evaluation of the predicted no-effect concentration (PNEC). Moreover, other coefficients may be useful such as median lethal concentration (LC_50_) or median effective concentration (EC_50_). To study ecotoxicity of ILs, various biological tests are used, utilizing fishes, invertebrates, phytoplankton [128] inhabiting the aquatic environment. Some studies have evaluated ILs toxicity to the marine bacteria *Vibrio fischeri* [50], and their influence on the enzyme activities [109].

However, algae examinations are the most widespread because of its simplicity, affordability, and the leading role of algae in energy transfer [129]. Water quality—fresh water algal growth-inhibition tests with marine algae *Skeletonema costatum* and *Phaeodactylum tricornutum* (EN ISO, 1995), and another one with *Scenedesmus subspicatus* and *Selenastrum capricornutum* (EN, 1993) are recommended by the European Commission Standardization (CEN), International Organization for Standardization (ISO, 1993, 1995) and Organization Economic Cooperation and Development (OECD, 1993).

It has been proven that the environment or rather current circumstances are capable of modulating the adverse biological effects of ILs. In 2005, Latała et al. [116] examined the acute effect of selected imidazolium-based ILs on the green alga *Oocystis submarina* and the diatom *Cyclotella meneghiniana* inhabiting the southern Baltic Sea. While *O. submarina* acclimatized to the lower concentrations of ILs after ca. 5 days of exposure, the growth of *C. meneghiniana* was effectively inhibited. The authoress observed, additionally, that at higher salinities, the toxicity of 1-butyl- and 1-hexyl-3-methylimidazolium entities towards *O. submarina* was significantly lower than noted at low salinities. The obtained results show the influence of salinity fluctuations on algae toxicity. The highest toxicity to all tested organisms is observed in freshwater and then decreases with increasing salinity. At the same concentration of ILs the toxicity decreased eightfold with algae and approximately three times in cyanobacteria within a certain salinity range from 0 to 32 practical salinity units (PSU, expressed as parts per thousand) [62]. The obtained results suggest that the toxic effect of ILs on algae can be reduced by increasing the salinity.

In 2008, Yun et al. described the effect of imidazole ILs with different anions on ecotoxicity in relation to *Selenastrum capricornutum* algae, which live in surface waters. The authors mainly assessed the influence of the anionic component of ILs. The obtained results showed that *Selenastrum capricornutum* was most sensitive to polyfluorinated anions such as [SbF_6_] and [PF_6_] [117].

Invertebrates are important elements of aquatic environments. As the core of the food chain, they exert a profound effect on the functioning of the whole ecosystem. That is why they are common models for ecotoxicity studies. A lot of research on IL toxicity has been conducted on *Daphnia Magna*. Experiments performed on the basis of imidazole and pyridine cations and quaternary ammonium anions have confirmed the correlation between the length of the alkyl chain and the toxicity of ILs. Additionally, it was noticed, that the number of aromatic nitrogen atoms harmfully influences this living organism [94]. Another invertebrate living in an aqueous ecosystem is a snail–*Physa acute*. The experiments have proven that this snail is sensitive to IL concentration ranges between 3.5 and 1800 μM [48].

The toxicity of ILs to vertebrate animals can help to learn about the effects and real damages that these compounds may cause to the environment. The most common model to study the influence of ILs on vertebrate organisms is the zebrafish (*Danio rerio*). It has been proven, that vertebrates are less vulnerable to the influence of ILs than invertebrates [48]. It has been observed that imidazolium-, pyridinium-, pyrrolidinium-based ILs are not toxic to fishes. Surprisingly, ammonium salts appeared to be more toxic. Moreover, the potential immunotoxic activity has been proven based on the activity of lysozyme in different organs of *Cyprinus carpio*. For 1-methyl-3-octyl imidazolium bromide [OMIM][Br] inhibition of the immune system has been observed after 7 days of exposure to IL at a concentration of 300 mg L^−1^ [130]. Amphibians (*Rana nigromaculata*) are also used to study the toxicity of ILs in an aquatic environment. In this case, the presence of ILs in water causes an increase of embryo mortality and a visible decrease in the weight of fetuses and potential teratogenic influences [48].

It is worth noting that different aquatic organisms exhibit varied sensibility depending on the kind of IL tested [62]. For instance, [1-butyl-3-methyl-Py] [Br] exhibited toxicity with EC_50_ = 2884 µM toward *Selenastrum capricornutum,* whereas regarding *Vibrio fischeri,* the EC_50_ value was much higher of 4677 µM [60]. Considering toxicity of ILs towards *Selenastrum capricornutum*, they can be arranged in the following order: [1-butyl-3-methyl-Py] [Br] with EC_50_ =2884 µM < [1-butyl-3-methyl-MIM] [Br] with EC_50_ =1047 µM. Regarding to *Vibrio fischeri,* [1-butyl-3-methyl-Pyr] [Br] was even less toxic with EC_50_ =4677 µM [32]. The algae *Scenedesmus vacuolatus* and the plant *Lemna minor* were more vulnerable to [1-butylo-1-metylo-Py] [Cl] than the bacterium *Vibrio fischeri* showing EC_50_ equal to 210 µM for the marine plant, 2340 µM for *Vibrio fischeri,* and 390 µM for the algae [84].

## 4. The Importance of Ionic Liquids in Agriculture

The presence of toxic chemicals in the environment has a negative influence on biota. The eco-toxicity of ILs is a consequence of migration in groundwater; bioaccumulation in aquatic or terrestrial ecosystems [48,131,132,133,134] (Table 3). The degree of ecotoxicity of ILs on crops depends on numerous factors. The authors emphasize the influence of soil sorption capacity and the length of ILs alkyl chain [135,136]. The individual resistance of plants to all kinds of xenobiotics is also important [48,137,138,139,140]. An interesting effect was observed in independent studies on *Scenedesmus vacuolatus* and wheat seedlings (*Triticum aestivum)* by Matzke et al. [141] and Zhang et al. [142] who proved the synergistic interaction of imidazolium ILs with cadmium cations.

The real threat is the penetration of ILs into the soil environment, where, absorbed by soil colloids, they will have an impact on the development of cultivated plants as well as the edaphon, ie all living organisms living in the soil [48,53,54,66,136,137,138,139,140,150,151]. Sorption of ILs onto soil was the aim of several studies [135,136,152,153,154] which showed that it is the imidazolium and pyridinium cations with long-chain hydrophobic substituents that can adsorb easily onto various kinds of soils becoming persistent contaminants in the environment. In turn, short alkyl-chains substituents possessing additionally polar functional groups forming more mobile hydrophilic ILs underwent unrestricted transport through soils causing danger of groundwater contamination [135,153]. It turns out that soil colloids, due to their negatively charged surface, easily bind imidazole or pyridinium cations, contributing to the inhibition of phytotoxicity [138,140]. Moreover, the increase in the amount of organic carbon in soil contributes to the reduction of the toxicity of ILs [53,138,151].

Stepnowski et al. [135] proposed a two-layer sorption model to assess ecotoxicity. According to this model, the first layer of the IL adheres directly to the soil surface due to electrostatic forces, and the second outer layer is formed by hydrophobic interactions. This model was confirmed experimentally by Matzke et al. [138] who assessed the phytotoxicity of 1-ethyl, 1-butyl, 1-octyl-3-imidazoliums on the development of cress and wheat. The authors observed that the degree of toxicity of ILs increases proportionally with the elongation of the alkyl chain up to only 4 carbon atoms. They interpreted the obtained result on the basis of the greater mobility of ILs with shorter side chains.

There is only a little data available on the biodegradability of ILs by microorganisms in the soil. In 2006, Kumar et al. [155] investigated the degradation of 1-butyl-3-methyl imidazolium tetra-fluoroborate by *Pseudomonas putida* and *Escherichia coli*. Modelli et al. [156] examined biodegradation of two imidazolim-based ILs, namely 1-butyl-3-methylimidazolium and 1-methoxyethyl-3-methyl-imidazolium with the BF_4_ and N(CN)_2_ anions. The observed biodegradability ranged from 17% to 52%. Steudte et al. [157] pointed on the poor stability of common IL anions such as [N(CN)_2_], [C(CN)_3_], [B(CN)_4_], [(CF_3_SO_2_)_2_N], [(C_2_F_5_)_3_PF_3_], [H(C_2_F_4_)SO_3_] in a strong acidic and basic environment. Thus, different biodegradation stimulation methods covering various biotic and abiotic parameters should be tested to resolve this problem.

In 2009 [140] Studzinska and Buszewski examined the sensitivity of *Lepidium sativum L*. germination to imidazolium ILs in aqueous solutions and soils artificially contaminated with ILs. An almost linear relationship between the decrease in root germination and the amount of IL uptake was observed. Furthermore, the influence of carbon content in soil on the toxicity of ILs was evaluated. The authors concluded that the negative effects of ILs were more pronounced with the decrease of total organic carbon quantity in the soil.

Invertebrates living in soil ecosystems are rarely considered a measure of cytotoxicity. An important example is earthworm (*Eisenia fetida*). The toxicity tests of imidazole IL performed on this organism revealed the following issues: the growth decrement ranged between 8.5 and 42.5% depending on the concentration of the IL, 55% decrease in reproduction, inhibition of the activity of ATPase in the presence of the IL (1–5 mg/kg of the soil) [143].

Currently, ILs are considered as potential substitutes for pesticides thanks to their antibacterial and fungicidal properties [150,158,159,160]. Intensive research is also conducted on the use of ILs as herbicides and plant growth regulators [113,161,162]. The structure of herbicidal ILs (HILs) is characterized by at least one ion with confirmed herbicidal properties [163]. Therefore there is a need to also test ILs in terms of phytotoxicity [164].

## 5. Usefulness of Ionic Liquids in Medicine

ILs have been tested for antibacterial and antifungal activities. Most of them exhibited activity against Gram-positive and Gram-negative pathogenic bacteria, which paves the way for using them as chemotherapeutic agents. It has been proven that imidazolium-based ILs are inserted into the bacterial plasma membranes changing their properties, in this way [165]. Several reports have also described the anticancer activity of ILs [166,167,168,169]. In 2017, Dias et al. [170] collected works describing the recently proposed applications of ILs as antitumor agents. Moreover, owing to their structural tailoring, they may be placed in aqueous, oily, or hydroalcoholic solutions [69,72], which is important when considering their use as excipients in many drug delivery systems [171].

### 5.1. Toxicity of ILs Against Human Cells (Cytotoxicity)

Cytotoxicity assays utilize different cell cultures for a screening risk evaluation. The influence of ILs on the cytotoxicity has been studied on different cell lines (Table 4). The most common parameter used as a metric of cytotoxicity is IC_50_ value. IC_50_ represents the concentration of the substance at which 50% of the cells are able to live. The higher value of the IC_50_ is connected with the lower the cytotoxicity of the examined substance [172].

Epithelial cells have been frequently chosen as they are the most directly exposed to contact with toxins. Stepnowski et al. studied the toxicity on HeLa human tumor cell lines that represent prototypical human epithelium cells [52]. The authors compared EC_50_ values obtained for investigated imidazolium ILs to chosen organic solvents, e.g., dichloromethane, phenol, xylene, and ethanol. Surprisingly, the ILs EC_50_ values were 4–5 times lower.

Many studies are conducted on other tumor cell cultures. For instance, Ranke et al. [50] applied a promyelocytic leukemia rat cell line and a rat glioma cell line, to examine the toxicity of imidazolium-based ILs with [BF_4_], [PF_6_], and [Cl] anions. The performed tests confirmed higher toxicities of the lipophilic ILs with long length alkyl chain, the negligible influence of anion kind on toxicity.

More precisely, the influence of the anion has been studied using the IPC-81 rat leukemia cell line by Ranke et al. [147]. The authors confirmed the almost negligible cytotoxic influence of almost all studied anions with the exception of some of them mostly polyfluorinated ions such as [SbF_6_], [Co(CO)_4_], [N(CF_3_)_2_].

In 2017, Salminen et al. [173] estimated the cellular toxicity or cytotoxicity of a variety of piperidinium and pyrrolidinium bromides. It appeared that the cytotoxicities of ILs assessed using the human breast cancer cell line MCF7 were similar to those of imidazolium ILs [50,184], and increased with an increase in the alkyl chain length. The lowest measured toxicity expressed by IC_50_ (mM) was 43.40 observed for [MPPyrr][Br]. However, it was still almost 2-fold higher in comparison to NaBr for which IC_50_ was 94.6. A comparison of obtained results with literature data proves the fact that piperidinium and pyrrolidinium are less toxic than their pyridinium homologues [185]. Moreover, the authors indicated that [Tf_2_N] anion causes increased toxicity compared to bromides for most studied salts.

To estimate the toxicity of ILs, enzyme inhibition assays have also been reported. Stock et al., applying structure-activity relationship (T-SAR), have found that a longer alkyl chain resulted in a stronger inhibition of the acetylcholinesterase [86]. In turn, Stepnowski’s group investigated the inhibition of AMP deaminase by imidazolium-based ILs [110]. The authors emphasized the predominant role of the cation type on the toxicity of ILs.

In 2018, Sommer et al. [186] performed a systematic analysis of 27 different ILs, with the aim to determine their effect toward viruses, bacteria and enzymes (two non-enveloped, one enveloped virus: P100, MS2 and Phi6, two Gram negative and one Gram positive bacteria: *E. coli*, *P. syringae* and *L. monocytogenes*, one enzyme: Taq DNA polymerase.

The obtained results showed that some ILs were virucidal, and they exhibited the effects on enveloped viruses, bacteria, and enzyme inhibition. However, an investigation performed using structure-activity relationships (SARs) did not identify specific structural moieties of ILs, which were responsible for ILs activity.

The mechanism of (cyto) toxicology of ILs has still remained unclear. Jing et al. [187,188], have investigated the interaction of ILs possessing amphiphilic characteristics with a lipid bilayer as a model cell membrane to examine cytotoxicity at a molecular level. By the use of fluorescence imaging and light and X-ray scattering, they have found that ILs disrupt the lipid bilayer by insertion of IL, end-capping the hydrophobic edge of the lipid bilayer. The insertion of ILs causes the swelling, and finally disintegration of the lipid bilayer. These interactions showed evident dependence on the hydrophobicity of IL cationic alkyl- chain and anions.

### 5.2. Ionic Liquids as Active Components of Pharmaceutical Formulations

Around 50% of drugs are used in the form of salts. That is why by pairing appropriate ions, the required drug properties can be finely tuned. In production processes and the pharmacokinetics of drugs, there are some important drug characteristics such as the melting temperature and solubility [189]. However, the most significant problem in the pharmaceutical industry appears to be the crystal polymorphism in pharmaceutical compounds [190,191,192,193], which may convert an effective dose into a lethal or an ineffective dose by changing the solubility of the active ingredient [194].

Other inconveniences connected with the solid form of drugs besides polymorphic conversions, have been reported such as poor solubility and a decrease of bioavailability. That is why, the pharmaceutical industry is still interested in looking for new drug forms, such as salts, solvates, and co-crystals [166,190], or active liquid forms with the aim to avoid the possibility of polymorphism problems.

The therapeutic efficiency and bio-accessibility of a drug are generally restricted by the low solubility or low permeation of drugs through semipermeable membranes. This may lead to incorporate higher drug doses to reach the therapeutic effect [195]. In this context, ILs represent an alternative to traditional drug delivery systems to enhance solubility and stability. Recently published studies have proven that several ILs improve the shelf life of proteins in solution, retaining their properly folded structures [196]. That is why they appear to be promising stabilizing solvents for protein therapeutics [197,198,199,200]. ILs are able to replace the water in protein-based pharmaceutical formulations for parenteral injection substituting costlier lyophilized products. Properly designed components can provide appropriate polarity, viscosity, hydrogen-bonding capacity, and conductivity.

Since some researchers have proven the negative influence of ILs for the growth of bacteria and fungi, we can utilize these for eliminating pathogenic microbes resistant to antibiotics and other medicines [201,202]. There are many examples of using ILs in medicine. They have been used for disinfection as disinfectant detergents [158] or additives for dressing materials. Furthermore, they became components of drugs of the so-called retarded action. There are also preliminary, very promising results of using ILs in tumor therapy [137,203,204].

The application of ILs into the biosciences has been delayed by their questionable safety. However, the toxicity of ILs observed toward microorganisms has led to the proposal for the use of some of them [70,166,205,206] in new bioactive materials as antiseptics [42,207,208,209]. For instance, ILs based on ammonium and benzalkonium cations with saccharinate and acesulfamate anions show very promising bactericidal and fungicidal activity, especially against *Streptococcus mutans*. Some of ILs possess broad-spectrum activity against a variety of microbial pathogens, such as methicillin-resistant *Staphylococcus aureus* (MRSA) [166]. A very promising application of ILs concerns their activity against microbial biofilms. Microbial biofilms contain different bacteria, such as MRSA, growing in colonies adherent to various surfaces. A dangerous feature of biofilms is tolerance and resistance to antibiotics [209] Proper selection of ILs’ components could facilitate drug entry into the biofilm.

Another problem concerning drug bioavailability is difficulty in crossing biological membranes caused by too high hydrophilicity. The correct selection of an active ion and combining it with a lipophilic one could solve this problem. An example illustrating this approach is lidocaine docusate. This is an IL containing the local surface anesthetic lidocaine cation and the hydrophobic docusate anion, (a dispersing agent) shown to be absorbed in epithelial intestinal cells. Owing to the above connection the final product is less soluble in water and could retain longer on the surface of the skin ensuring slow-release mechanism of action and providing greater antinociception [3,166].

Recently, a few combinations of two active units (APIs) in the singular drug forming so-called “dual functionality” have been produced. In 2007 Pernak et al. [210] reported a method for the preparation of ILs combining ions of varied activity with nutritional and energetic ingredients. In literature, numerous examples of pharmaceutically active salts prepared by connecting an active ion with a simple and inert counterion have also been reported. However, many other drugs (or their APIs) could be used for the preparation of pharmaceutically active ILs. Some of them could be used as the cation unit, such as omeprazole used to treat gastroesophageal reflux disease. Some of them are able to form the anion unit, such as the amoxicillin antibiotic. Numerous drugs like the antiepileptic gabapentin possess dual functionalities. The angiotensin converting enzyme inhibitor- lisinopril, which is used for hypertension [5,211].

To apply ILs as components of drugs requires a systematic evaluation of the toxicological properties [56], and evaluation of their cytotoxicity [54,212]. Furthermore, using ILs as components of the parenteral formulations, the osmotic strength has to be also exactly defined, since, in the case of ILs, full ionization cannot be assumed. Besides, ILs are able to create ion pairs in aqueous solutions [213].

Considering the safe use of these drugs in vivo, choline and phosphates were selected as starting materials due to their natural prevalence in the human body. However, choline-based ILs have been proven to exhibit varied toxicities starting from low one as for [CDHP] to high as for [CTMP] and [CBEH] [212]. In 2010, Elliott [179] evaluated the cytotoxicity of choline-based ILs containing phosphate and phosphinate anions with the purpose to identify the least toxic composition as potential components in pharmaceutical formulations. The authors observed that the anion has a great effect on the osmotic coefficient of the solution. For instance, [CDHP] ensured an osmotic coefficient of 1.0, whereas [CBEH] achieved an osmotic coefficient of 0.3. Thus [CBEH] was much more like to molecular solvent and can be used to minimize osmolality of parenteral preparations. It has been already proven that choline dihydrogen phosphate [CDHP] applied as the co-solvent is able to improve the shelf life of cytochrome c, retaining either its structure or activity [199,214,215]. In 2018, [216] two choline-based ILs, namely (2-hydroxyethyl)-trimethylammonium methionate [Chol][Met] and (2-hydroxyethyl)-trimethylammonium ((*R,R*)-3,3′-ditiobis(2-aminopropanoate) [Chol][Cys], were used to evaluate their influence on the solubility of caffeine as a model hydrophilic drug. Results showed that the solubility of caffeine increased from 20.02 ± 0.06 mg/mL in deionized water to 22.21 ± 0.91 mg/mL with [Chol][Cys] and 24.74 ± 1.01 mg/mL with [Chol][Met]. The authors suggested that the choline-based ILs could be used as functional ingredients in drug delivery systems suitable for enhancing solubility of other hydrophilic drugs. The use of ILs as functional excipients to overcome poor drug solubility has been proposed recently by Júlio et al. [217]. The authors proposed efficient, non-toxic IL-polymer nanoparticle hybrid systems composed of choline-based ILs and poly (lactic-co-glycolic acid) [PLGA] 50:50 or 75:25 to load rutin into the delivery system. Examples of ILs with pharmaceutical potential are collected in Table 5.

## 6. Design of Environmentally Harmless ILs

Since possible combinations of ions in ILs reach millions, it is very important to elaborate rules suitable for design environmentally harmless ILs. Changing both the cation and anions structure provides the change of lipophilicity, miscibility with water which is linked to further biodegradability and toxicity to living organisms. By testing the toxicity of already existing ILs, the factors responsible for toxicity can be identified, and it will be possible to plan the synthesis of new classes of ILs that are safe for the environment.

The toxicity of ILs depends on a number of factors such as the structure of the cation, the length of the alkyl chain, the concentration, and, finally, the specific resistance of the organism. Structure-activity relationships (SARs) appears to be a rational strategy for the design of new green ILs as well as for predicting their toxicological and ecotoxicological properties [222].

### 6.1. Structure-Activity Relationship

**Figure 1:**
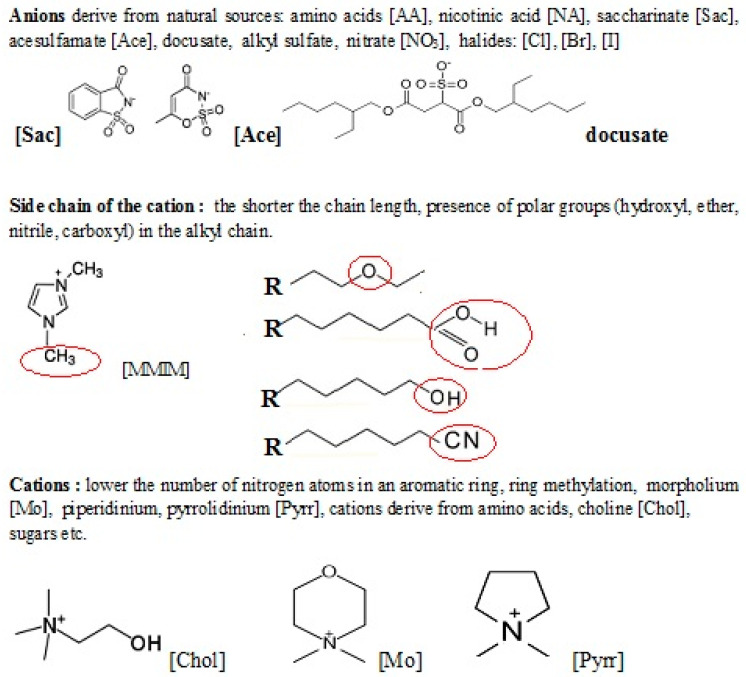
Chosen substructures of ILs influencing their toxicity.

To consider the toxicity of ILs, we have to take into account three individual substructures: (i) a positive moiety known as the head-group, (ii) the substituents on the head-group, and (iii) the anion [40] (Figure 1). So far, the predictions utilizing the t-SAR approach have mainly taken into account the length of the alkyl chain and the type of the head group. Many authors emphasize the importance of the cation structure as the most important factor influencing the toxicity of ILs [48,53,54,66,74,222]. In 2007, Stolte et al. [53] estimated the toxicity of ILs containing several cations: dimethylpyridinium, pyridinium, imidazole, piperidinum, pyrrolidinium, morpholinium and ammonium cations against *Vibrio fischeri, Scenedesmus vacuolatus, Lemna minor*. The study showed that the most toxic were the ILs with aromatic cations, especially dimethylpyridinium, compared to quaternary ammonium salts. The toxicity of imidazolium and pyridinium-based ILs for enzymes, microorganisms, cells, and even whole animals and plants has been confirmed by others [48]. Furthermore, these ILs, in most cases, appeared not biodegradable [132]. In contrast, ILs containing quaternary ammonium or alicyclic cations such as morpholium, piperidinium or pyrroliudinium, exhibit lower toxicity in comparison to those with aromatic cations such as imidazolium and pyridinium [197]. There are some pieces of evidence that the presence of polar hydroxyl, ether, and nitrile groups in the alkyl chain may minimize the toxicity of ILs to an enzyme such as acetylcholinesterase (AChE) [222].

It has been proved that the toxicity of ILs depends also on the length of the side chain. It has been demonstrated that the longer the *n*-alkyl chain length of the ILs is responsible for higher toxicity [48,53,54,66]. In 2007, Matzke et al. [54] tested the toxicity of 1-alkyl-3-methylimidazole against marine organisms such as marine bacteria (*Vibrio fischeri*), algae (*Scenedesmus vacuolatus*), duckweed (*Lemna minor*), and terrestrial such as wheat (*Triticum aestivum*) and watercress (*Lepidium sativum*). It turned out that regardless of the type of anion, the elongation of the alkyl chain has a destructive effect on the viability of organisms. These results are confirmed by numerous published works [140,167,223,224,225,226,227]. The mechanism of toxicity of ILs is probably related to the dysfunction of the lipid bilayer, which leads to cell lysis [53,66].

This tendency of increasing toxicity with an increase in the length of the alkyl chain has been observed by numerous authors. Pernak et al. observed increased antimicrobial activity with increasing alkyl chain length on pyridinium salts, imidazolium, and quaternary ammonium salts [167,224,225,226,227]. Similar activity was shown by alkylimidazolium and alkylpyridinium salts paired with anions such as [BF_4_], [PF_6_], [C] anions [49,50] against the marine bacterium *Vibrio fischeri.* A similar trend was observed in mammalian cell cultures [50], human cell HeLa18 [52], and higher organisms, including soil nematodes *Caenorhabditis elegans* [147] and freshwater snail *Physa acuta* [228], *Daphnia magna* [119]. Most studies confirm that the type of anion has a minimal effect on toxicity [49,50,52,147,224] although numerous authors point out some contribution of the anion to the toxicity [228]. The situation is different when the counter-anion belongs to the polyfluorinated ion group. In this case, significant toxicity was observed [52,123,212] which was due to anion hydrolysis leading to the formation of toxic fluorides.

In 2005, Maginn et al. [51], proposed a statistical model to predict the toxicity of the imidazolium, pyridinium and quaternary ammonium-based ILs for two aquatic organisms *Vibrio fischeri* and *Daphnia magna*, based on their quantitative structure-property relationships (QSPRs). Data to build the model included molecular-based descriptors, and microbial toxicity estimated using a standard Microtox. The authors applied the following parameters to measure toxicity: the lethal concentration (LC_50_) which is the concentration of a chemical that causes death to 50% of the test organisms, and EC_50_ parameter representing effective IL concentration at which organism’s respiration was reduced by 50%. The relation between logEC_50_ predicted by the model and values measured for *Vibrio fischeri* achieved very good reproducibility with an R^2^ = 0.782. In turn, the logLC_50_ data for *Daphnia magna* are reproduced with an R^2^ of 0.88. Considering the cation type, the trend of increasing toxicity was observed in the following order: ammonium < pyridinium < imidazolium < thiazolium < tetrazolium. Furthermore, besides the well-recognized relation between toxicity and alkyl chain length, the elaborated models predict that toxicity can increase with the number of nitrogen atoms in an aromatic cation ring, whereas a decrease of ring methylation as well as with an increase in the number of negatively charged atoms on the cation cause inhibition of toxicity. Authors also confirmed that the anion plays a meaningless role in the toxicity of the studied ILs—however, the positively charged atoms on the anion could unexpectedly increase toxicity.

In 2007, Matzke et al. [54] applied an effective strategy of the flexible (eco)toxicological test battery considering aquatic and terrestrial compartments as well as different trophic levels to uncover hazard potentials of ILs. The authors systematically analyzed the anion kind ([Cl], [BF_4_], [(CF_3_SO_2_)_2_N], [(CF_3_)_2_N], octylsulfate, bis(1,2-benzenediolato)borate), and the length of the cation substituents on ILs’ toxicity. The authors have confirmed the side chain length effect, however, for the tested anion moieties, the results were inconsistent. The greatest (eco)toxicological hazards were ILs containing [(CF_3_SO_2_)_2_N] as an anion for which a clear harmful effect on the environment appears to be evident.

The examinations of activities and properties of ILs are cost and time-consuming, that is why some studies have been performed by the theoretical calculations based on quantitative structure-property/structure/activity relationships (QSPR/QSAR). The QSPR model has been proposed for the evaluation of the melting temperature of the imidazolium ILs [229]. The quantitative structural-activity relationship studies (QSAR) have been used for predicting the toxicity against human HeLa and MCF-7 cancer cell lines [230], the leukemia rat cell line (IPC-81) [231,232]. Zhu et al. [233] elaborated quantitative structure-activity relationships (QSAR) models using an extreme learning machine (ELM) algorithm to evaluate the toxicity of ILs toward the acetylcholinesterase enzyme.

### 6.2. ILs Composed of Renewable Biomaterials

Due to the high stability of ILs, the risk of their accumulation in the environment cannot be ignored. Wells et al. studied the potential of ILs to the biodegradation using biochemical oxygen demand in five days (BOD_5_) [234]. It appears that imidazolium, pyridinium, phosphonium, and ammonium-based ILs do not undergo biodegradation. Other reports also confirmed the absence of [BMIM][PF_6_] biodegradation [115]. Therefore, nowadays, the interest of the scientific community in biodegradable and biorenewable ILs can be observed [235]. To produce a new class of virtually green ILs, ions are obtained by modification of natural sources, for instance, biodegradable surfactants [46]. It has been shown that ester functionality is able to enhance biodegradation of ILs owing to higher susceptibility of the ester group to enzyme hydrolysis [236]. Furthermore, the biodegradability improvement can be achieved by adding a methyl group to the 2-position of the imidazolium cation and octyl sulfate as counter anion [237].

Considering the high stability of ILs as waste products, it is very important to find adequate disposal and degradation methods. So far, some methods of degradation have been tested such as advanced oxidation processes [238,239,240,241,242,243,244,245,246], and biodegradation [46,114,240,247,248,249], and achieved the degradation of ILs by combining UV radiation with catalytic oxidants using hydrogen peroxide and titanium dioxide. However, more hydrophobic ILs with longer alkyl substituent and phosphonium-based head-group appeared to be more resistant to biodegradation. Li et al. [240] proposed very efficient oxidative degradation using a mixture of hydrogen peroxide/acetic acid under ultrasonic activation. In turn, degradation by oxidation in the Fenton system (1 mM Fe(III) and 100 mM H_2_O_2_) of imidazolium, and pyridinium based ILs assured efficiency on the level of 68–97% [82,242,243]. More sophisticated methods have been also tested such as hydrothermal mineralization with Ca(OH)_2_, and a photocatalytic decomposition method [239] and electrochemical oxidation on a boron-doped diamond (BDD) anode [250]. The biological methods were not so efficient because of microbial breakdown [248].

Since Pernak pointed out the problem of ecotoxicity and of ILs [225], a new class of biodegradable ILs has gained more attention in the scientific and industrial community [132,140]. The new generation of biodegradable ILs is created by combination of ions derived from natural α-amino acids, α-amino acid ester salts [251], or saccharin and acesulfame used as non-nutritional sweeteners [252]. Improvement of bio-renewable and biodegradable properties of this new class of ILs has been achieved by the addition of nontoxic inorganic [NO_3_] or organic saccharide anions [253].

According to this trend, cholinium, being a quaternary ammonium with a polar hydroxyl group, has been chosen as the IL cation of relatively low toxicity, undergoing readily biodegradation [179,254,255,256,257]. These ILs are synthesized from renewable starting materials by the use of neutralization reactions. Therefore, deep eutectic solvents are sometimes referred to as the new generation of ILs, despite the fact that a eutectic mixture is not composed of ions. Nowadays, DESs seem to be a less toxic alternative to ILs. Abbott and co-workers [258] discovered that choline chloride can form “deep eutectic solvents” with hydrogen-bond donors. For example, at an appropriate molar ratio of urea to choline chloride (2:1), an ion-liquid like solvent can be obtained, which is a liquid at room temperature [29].

It was observed that an exchange of the cation from imidazolium-based to cholinium strongly lowered the toxicity to the brine shrimp *A. salina* and HeLa cell culture ten times. However, this relation has not been confirmed in tests on bacteria [69]. Choline chloride (2-hydroxyethyltrimethyl ammonium chloride or vitamin B_4_) is a useful and cheap precursor for the preparation of ILs. Although it has a high melting point (298–304 °C), new ILs can be made by metathesis reactions by replacing the chloride anions by other counterions. Furthermore, by combining these ions, which are derived from an essential vitamin-like nutrient and artificial sweetener, one could obtain the product classified as the “food grade” IL. The ecotoxicities of choline saccharinate and choline acesulfamate were evaluated using a standard assay with the invertebrate *Daphnia magna*. The obtained results show that choline-based ILs were approximately two orders of magnitude less toxic than imidazolium or pyridinium ones. Till now, a variety of cholinium containing ILs have been described [259,260].

In 2007, Nockemann et al. [255] described the synthesis of two hydrophilic ILs with low (eco)toxicity; namely choline saccharinate and choline acesulfamate, prepared from easily available starting materials (choline chloride and a non-nutritional sweetener).

In 2010, Pereira et al. evaluated the toxic effect of the anion in a series of cholinium-based ILs [256]. The toxicity of these ILs was evaluated using filamentous fungi as the model eukaryotic organisms. The authors observed the active growth of the tested species in media containing a high concentration of IL, even up to molar ranges. The influence of the anion structure on the overall toxicity was related to the length of the hydrocarbon chain and was ranked in the following order: ethanoate < propanoate < butanoate < pentanoate < hexanoate < octanoate < decanoate. As the anionic components of biodegradable ILs, amino acids (AAs) have been often proposed owing to their structural diversity, and widespread abundance in nature [40,261,262]. Zong’s group described synthesis cholinium-based ILs with amino acids as the anions ([Chol][AA]) useful for selective lignin removal from lignocellulosic biomass [263,264,265,266], and for organic synthesis as catalysts [259,260]. Moreover, there are some examples of amino acid-based ILs, in which amino acids are both the cations and anions [251,252,262,263,267].

It has been proved that functionalized guanidinium ionic liquids showed non-toxic behavior toward the human colon carcinoma CaCo-2 cell line [268]. Performed biodegradability tests with bacterial cultures *Pseudomonas putida* and *Bacillus subtilis* and a closed bottle test showed that tetramethylguanidinium [TMG] cations with benzoate, salicylate, lactate, dihydrogen phosphate, nitrate, formate, acetate, propanoate, butanoate and valerate anions are also easily biodegradable [269].

Although MILs are not applied at the industrial scale, their environmental impact is being constantly evaluated. Sintra et al. [270] examined the ecotoxicity of cholinium salt derivatives with magnetic properties. The obtained results proved that all MILs tested are moderately toxic, or toxic toward the luminescent bacteria *Vibrio fischeri*. Similarly to imidazoilium-based ILs, the toxicity of MILs is related to the length of the alkyl side chain connected to the cation, the number of hydroxyethyl groups, and the kind of the metal in the anionic part. A magnetic anion, namely [MnCl_4_]^2−^ was established to be the least toxic.

Several works described the synthesis of ILs from renewable reactants such as nicotinic acid [266], and sugars [271,272]. Feder-Kubis [273] used plant sources as the starting materials to synthesize new ILs. Engineered ammonium and imidazolium salts were obtained using the (1*R*,2*S*,5*R*)-(−)-menthol moiety and benzo-[1.2.3]-thiadiazole-7-carboxylate counterion. Obtained ILs exhibited a high level of antimicrobiological activity, higher than benzalkonium chloride, which is commonly used in biocides, and at the same time inducing efficiently plant resistance by combining them with the antibiocidal agents. Owing to the proposed strategy it was possible to prepare bifunctional salts connecting benefits of ILs like ionic characteristics, solubility in water, with additional antibacterial. Feder-Kubis et al. described the synthesis of ILs containing bicyclic monoterpene moiety derivatives of (1*S*)-*endo*-(–)-borneol and (1*R*)-*endo*-(+)-fenchol [274]. The ILs based on monoterpene derivatives namely, (1*R*,2*S*,5*R*)-(–)-menthol or bicyclic (1*R*)-endo-(+)-fenchol have been recognized as the potential antifungal agents. Their antifungal activity consisting of the selective destabilization of the fungus membrane was studied using a Langmuir monolayer mimicking fungal (ergosterol) and mammalian (cholesterol) membranes [275].

## 7. Closing Remarks and Future Perspectives

For the past years, ILs have been considered promising alternative to toxic organic solvents. This belief has been supported by some properties of ILs, such as their non-flammability, negligible vapor–pressure (10^−11^–10^−10^ mbar) and thermal stability (ILs decompose in the range from 250 °C to 450 °C). However, the above assumption appears to be partially wrong considering the fact that ILs are not a monolithic group of compounds. An example may be the observed flammability of imidazolium, pyridinium, or phosphonium-based ILs [43] or the thermal decomposition of 1-butyl-3-methyl imidazolium bromide at a temperature below 200 °C reported by Meine et al. [276]. Application of ILs in different areas of science and industry such as organic synthesis, catalysis, electrochemistry, analytical chemistry, nanotechnology has improved the efficiency and productivity of many processes owing to among others their recycling possibilities. Several authors have described the efficient reuse of ILs after a few consecutive cycles [277]. The widespread use of ILs increases the danger of these compounds being transferred to the environment. Therefore, the determination of the ecotoxicity of newly synthesized ILs with potential industrial use should be mandatory regardless of any declared green properties, whose perception has radically changed in the last years. Many of the studies presented in this review report the harmful effects of ILs on the environment.

The growing development of green technology demands the design of new, biodegradable materials [278,279,280]. That is why it is not enough to design an optimal IL for a specific purpose. Regarding the principles of green technology, we are designing ILs that are safe for the environment and do not liberate hazardous substances capable of killing living organisms. A rational guide for the synthesis of non-toxic biodegradable products from renewable sources such as amino acids, sugars, choline, bicyclic monoterpene moiety derivatives, etc. is urgently needed. 

## Figures and Tables

**Table 1:** Examples of studies on ILs’ toxicity against microorganisms.

Ionic Liquids	Microorganisms/other Subjects	Toxicity	Ref.
[BMIM][Br], [HMIM][Br], [OMIM][Br], [BMPyr][Br], [HMPyr][Br], [OMPyr][Br]	*Vibrio fischeri, Escherichia coli*, *Staphylococcus aureus*, *Bacillus subtilis*, *Pseudomonas fluorescens*, *Saccharomyces cerevisiae*	The toxicity towards *Vibrio fischeri* increases with an increase in alkyl group chain length. The log EC_50_ (ppm) 5, 10, 15 min. values reveal the trend of toxicity: [BMIM][Br] (~3.5) < [HMIM][Br] (~1.0) < [OMIM][Br] (~0.2); [BMPyr][Br] (~2.5) < [HMPyr][Br] (1.5) < [OMPyr][Br] (~0.3). The most significant antimicrobial activity was observed in tests with *B. subtilis*.	[49]
Alkyl (C_3_-C_10_) methyl-, ethyl-imidazolium with [BF_4_], [PF_6_], [Br], [Cl]	*Vibrio fischeri*/IPC-81 (leukemia cells), C6 (glioma cells)	The log EC_50_ (µM) values for *V. fisheri* was in the range from −0.182 to >−3.94. The ILs with the longest alkyl chain length showed higher toxicity.	[50]
[IM], [Py], [N]	*Vibrio fischeri*/*Daphnia magna*	The toxicity increases in the order: [N] < [Py} < [IM] < triazolium < tetrazolium, and decreases with ring methylation. Tested ILs’ concentration was from 0.001 to 100 mmol L^−1^. The log EC_50_ (mM) values for *V. fisheri* was in the range from −2.37 to >2.0.	[51]
[Py], [IM], [Mor], [Pip], [Pyr] [N], with halides, [(CF_3_SO_2_)_2_N]_2_^−^	*Vibrio fischeri*/green algae *Scenedesmus vacuolatus*, the fresh water plant *Lemna minor*	The non-aromatic compounds exhibited a lower toxicity (EC_50_ >10 000 µM) to *V. fisheri.* ILs combined with the [(CF_3_SO_2_)_2_N]_2_ were more toxic for *V. fischeri* than halides.	[53]
1-alkyl-MIM with [Cl], [BF_4_], [(CF_3_SO_2_)_2_N], [(CF_3_)_2_N], octylsulfate bis (1,2-benzene diolato) borate	*Vibrio fischeri/*enzymes-mammalian cells algae, wheat, cress, duckweed, a soil invertebrate.	Luminescence inhibition *V. fischeri* for 1-butyl-3-methyl-imidazolium ILs as EC_50_ (µM) changed depending on the anion in the order: octylsulfate (70) < [CF_3_(SO_2_)_2_N] (300) < [Cl[ (2500) < [(CF_3_)_2_N] (3000) < [BF_4_](3500).	[54]
[PMIM][Tf_2_N]	*Vibrio fischeri*/algae: *Pseudokirchneriella subcapitata, Chlorella vulgaris*, cladocerans: *Daphnia magna, Daphnia longispina.*	EC_50_ (mg L^−1^) 5 min. for *V. fisheri* was 480.0 after 15 min. of exposure it was 240.0, and after 30 min. decreased to 125.81.	[58]
[Bmim][PF_6_], [Hmim][PF_6_], [Omim][PF_6_]	*Lactobacillus rhamnosus* NBRC 3863	The survival rate of microbes expressed by the use of the amount of glucose consumed and CFU values were low in the presence of imidazolium-based ILs (half of the activity of the control group). A change of alkyl length in the imidazolium cation had little influence on the survival of the cells.	[67]
[IM], [Pyr], [Cho], with [Br], amino acids: arginine, cystine, glutamine, glutamic acid	*Bacillus subtilis, Escherichia coli/*crustacean *Artemia salina;* human HeLa cells	Toxicity was assayed by agar diffusion. Bacterial inhibition halo (cm) due to exposure to ILs was higher for *B. subtilis* than to *E. coli*. The toxicity of ILs to bacteria was smaller than that of chloramphenicol.	[69]
[EMIM], [BMIM], [HMIM], [Ch] with [Br], [TBI], [TBO], [TBT]	*Staphylococcus aureus, Enterococcus faecalis, Enterococcus hirae, Bacillus subtillis, Mycobacterium smegmatis, Escherichia coli, Pseudomonas aeruginosa, Klebsiella pneumonia, Candida albicans*	The antimicrobial activity was assessed by Microdilution method. Inhibition the growth of the microorganisms was measured after 24 h of incubation. *B. subtillis* (8 mm of inhibition zone for [HMIM][TBT], *P. aeruginosa* (9 mm for [BMIM][TBO], [EMIM][TBI]. Other imidazolium and the choline ILs showed no inhibition. [HMIM][TBT] and [EMIM][TBI] showed MIC values of 62.5 μg/mL against *B. subtillis* and *P.aeruginosa.* [Cho][TBO], [Cho][TBT] [Cho][TBI], [BMIM][Br], [BMIM][TBT] [HMIM][TBO] did not exhibit antimicrobial activity.	[72]
[P_6,6,6,14_][NTf_2_], [N_1,8,8,8_][NTf_2_]), [P_6,6,6,14_][Cl], [N_1,8,8,8_][Cl]	*Escherichia coli*	FT-IR spectra confirmed that ILs were accumulating within the cells. Chlorides were accumulated more rapidly than the biocompatible [NTf_2_][P_6,6,6,14_][NTf2] was accumulated in the membrane fraction.	[75]
guanidinium, phosphonium, [IM] with [Cl],[Br], [I], [CH_3_SO_3_^−^], [TOS]	*Vibrio fischeri*	The increasing toxicity with the increase in the alkyl chain length, the alkyl chains with ether or ester groups decrease the toxicity, the phosphonium- ILs were more toxic, the EC_50_ (mg L^−1^) 15 min, decreases in the order: [P_4441_][CH_3_SO_4_](237.60) < [P_4444_][Br](172.80) < [P_4444_][Tos](169.60) < [P_66614_][CH_3_SO_3_] (7.43) < [P_66614_][Cl] (7.10).	[76]
[BMIM]Cl	*Saccharomyces cerevisiae* AY93161	The single-cell morphology was unchanged, the reproduction rate decreased with the [BMIM]Cl concentration increasing. The EC_50_ and IC_50_ values were 0.53 and 0.39 g L^−1^ respectively.	[77]
[BMIM][PF_6_], [HMIM][PF_6_], [OMIM][PF_6_]	*Lb. rhamnosus* NBRC 3863, *Lb. homohiochi* NRIC 0119, *Lb. homohiochi* NRIC 1815, *Lb. fructivorans* NRIC 0224, *Lb. fructivorans* NRIC 1814, *Lb. delbruekii subsp. lactis* NRIC 1683*, P. pentosaceus* NRIC 0099, *Leu. fallax* NRIC 0210, *B. coagulans* NBRC 12,583	All lactic acid-producing bacteria could grow in the presence of imidazolium-based ILs. The number of viable cells of bacteria in the presence of imidazolium-based ILs (5% *v*/*v*) was lower than determined for control group, and decreased with the alkyl chain length.	[78]

Abbreviations: *Lb., Lactobacillus; P., Pediococcus; Leu., Leuconostoc; B., Bacillus;* LC_50_, the concentration of a chemical that causes death to 50% of the test organisms; FT-IR, Fourier transform infrared spectroscopy; CFU, the number of colony-forming units per milliliter of culture.

**Table 2:** Toxicity of ionic liquids to aquatic organisms.

Ionic Liquid	Marine Organisms	Estimated Parameters	Observations	Ref.
1-alkyl-MIM	*Daphnia magna, Vibrio fisheri*	EC_50_ (µM) 24 h; IC_50_ (µM) 24 h.	IC_50_ for *D. magna* ranged from 4 to 90 µM, and *V. fisheri* from 10 to 2200 µM.	[46]
[IM], [Py], [Chol], [P], [N] with [Cl], [Br], [BF4], [PF6], [NTf_2_]; dicyanoamide, diethylphosphate	*Daphnia Magna, Vibrio fisheri*	log EC_50_ (mM)24, 48 h; logLC_50_ (mM)24, 48 h.	ILs toxicity to *D. magna* ranged from the highly toxic 1-*n*-octyl-3-methyl imidazolium bromide (LC_50_ = 0.00004 mM) to the less toxic 1-*n*-butyl-3,5-dimethyl pyridinium bromide (LC_50_ = 0.097 mM).	[51]
[IM], [Py], [Mor], [Pip], [N] with [Cl], [Br], [N(CF_3_SO_2_)_2_]_2_	*Vibrio fischeri Scenedesmus vacuolatus, Lemna minor*	EC_50_ (µM)	For *V. fischeri* the non-aromatic ILs exhibited a low toxicity (EC_50_ > 10 mM), the aromatic ILs had EC_50_ > 1 mM, [N] < 10 µM. For *L. minor* EC_50_ for [N] > 1 mM, for *S. vacuolatus* [N], [Mor] ILs were no toxic.	[53]
[EMIM], [BMIM], [OMIM] with [Cl], [BF_4_], [8OSO_3_], [(CF_3_SO_2_)_2_N)], [(CF_3_)_2_N)], [(2-OPhO)_2_B]	*Vibrio fischeri, Scenedesmus vacuolatus*	EC_50_ (µM)	The EC_50_ values for *V. fischeri* ranged from 3500 [BMIM][BF4] to 70 [BMIM] [8OSO_3_]. The EC_50_ values for *S. vacuolatus* ranged from 840 [EMIM] [(2-OPhO)_2_B] to 25 [BMIM] [(CF_3_)_2_N)].	[54]
[BMIM], [HMIM], [OMIM] with [Br]	*Scenedesmus quadricauda*, *Chlamydomonas reinhardtii*	% of the growth rates, EC_50_ (mg L^−1^) 96 h	*S. quadricauda* (EC_50_ values of 0.005–13.23 mg L^−1^) *and C. reinhardtii* (EC_50_ values of 4.07–2138 mg L^−1^).	[55]
[BMIM], [BMPy], [BMPyrr], [TBA], [TBP] with [Br]	*Selenastrum capricornutum*	The percent inhibition of the growth rate (I %), LogEC_50_ (µM) 48, 72, 96 h.	LogEC_50_ 48 h ranged from 1.9 for [TBP][Br] to 3.67 for [BMPyrr][Br]; LogEC_50_96 h ranged from 3.02 for [BMIM][Br] to 4.09 for [BMPyrr][Br].	[60]
[EMIM], [BMIM], [HMIM], [OMIM], [DMIM] with [Cl]	*Oocystis submarina, Chlorella vulgaris*, *Cyclotella meneghiniana, Geitlerinema amphibium*	Growth inhibition [I %] in different salinities [PSU] (8, 16, 24, 32).	The 8 PSU salinity, caused growth inhibition from 60–70%, the further reduction for 16 PSU was the highest for *O. submarina* (c. 20–30%), for 32 PSU, the growth inhibition was 30–40% in *C.vulgaris*, *C. meneghiniana,* and 40–50% in *G. amphibium.*	[62]
[BMIM] with [PF_6_],[Tf_2_N],[BF_4_], [N(CN)_2_], [OTf], [NO_3_]; [BPyrr][Tf_2_N], [EMIM][OTs], [BPy][Tf_2_N], [BM_2_IM][PF_6_], AMMOENG 100, 110, 112, 130.	*Danio rerio*	LC_50_ (mg L^−1^)96 h, histological examination.	The imidazolium, pyridinium and pyrrolidinium showed a LC_50_ > 100 mg L^−1^, the ammonium salts showed LC_50_ in the range of 2.2 to 15.5 for AMMOENG 100 and 1.9 to 13.9 for AMMOENG 130. Hystological evaluation showed skin alteration represented by epithelial hyperplasia with single keratinocyte vesciculation and wide erosions together with disepitelialization of gill lamellae.	[63]
AMMOENG 100, AMMOENG 130, [BPy], [BMPyr], [BMIM], [C_2_ClMIM], [C_2_OHMIM] with [Tf_2_N]; [C_3_OHMIM], [HMIM], [HC_2_ClIM], [C_2_ClMIM], [C_2_(HIM)_2_]_2_ with [Cl]; [EMMor], [EBMor], [TMSiMMIM], [ETHT], [C_2_C_2_C_2_S] with [Br]; [Chol][PF_6_].	*Pseudokirchneriella subcapitata, Daphnia magna, Danio rerio*	EC_50_ (µM) 48, 72 h.	The toxicity decreases from pyridinium and imidazolium topyrrolidinium, ammonium, and morpholinium. A low toxicity characterized the sulfonium- and thiophenium-based ILs. The substitution of one or two carbon atoms of the longer alkyl chain with a more electronegative atom (chlorine or oxygen) reduced the toxicity.	[64]
[BPy], [HPy], [BMIM], [HMIM] with [Br]; [Chol] with [Gly], [Ala], [Phe], [Gln], [Met], [Arg], [Glu]; [BMIM] with [Ala], [Phe]	*Artemia salina*	mortality %, LC_50_ (mM) 24 h.	The IC_50_ values ranged from 0.079 to 0.114 for [IM], from 0.086 to 0.117 for [Py], and from 2.896 to 9.517 for [Chol] ILs.	[69]
[MMIM][CH_3_SO_4_] [EMIM][C_2_H_5_SO_4_], [BMIM], [HMIM], [OMIM] with [Cl]; [HMIM] [OMIM] with [PF_6_]	*Vibrio fischeri* (*Photobacterium phosphoreum*)	log EC_50_ (µM) 15 min; BOD_5_ (mg O_2_ L^−1^).	The EC_50_ values varied between 58,000 for [MMIM][MSO4]and 5 µM for [OMIM][PF6].	[74]
[BMIM], [P_4444_], [P_66614_] with [Tos], [CH_3_SO_3_]; [TMGC_4_], [TMGC_7_], [TMGC_12_], [(di-h)_2_DMG], [(C_3_O)_4_DMG],[C_10_C(O)OEMIM] with [I], [Cl], [Br].	*Vibrio fischeri*	EC_50_ (mg L^−1^) 5, 15, 30 min.	The EC_50_ (mg L^−1^) 15 min values were in the range 6.38–237.6 for [P], 653–735 for [IM], but for [C_5_O_2_MIM], [Cl] 40.55, 3.72–30.6 for [TMGC_n_].	[76]
[EMIM][BF4] [BMIM][BF4] [BzMIM][BF4] [HMIM][BF4]	*Oocystis submarina, Cyclotella meneghiniana*	The percentage inhibition of the cell growth.	the growth inhibition of O. submarina reached 80% for EMIM (500 µM) after 5 days; 60% to *C. meneghiniana; O. submarina* acclimatized to the lower conc. of ILs; the toxicity of ILs is reduced in 30% saline water.	[116]
[IM] with [SbF_6_], [PF_6_], [Cl], [BF_4_], [Br], [CF_3_SO_3_] [C_8_H_17_OSO_3_]	*Selenastrum capricornutum* (ATCC-22662)	The proportion of algal growth rate (OECD guidelines), EC_50_ (µM)96 h.	the EC_50_ values obtained ranged from 135 ([SbF_6_]) to 2884 mM ([Cl]).	[117]
[Pyr], [Pip], [Py], [IM] with [Br]	*Vibrio fischeri, Daphnia magna*	log IC_50_ (mg L^−1^)	The logIC_50_ values were the following: [IM]:1.219-3.55; [Py] 594-2.353; [Pip] 1.281-3.214; [Pyrr] 0111-2.715.	[118]
[BMPy], [HMPy], [OMPy], [OMIM], [HMIM], [TBP], [TBA] with [Br]; [BMIM][PF_6_]	*Physa acuta*	LC_50_ (mg L^−1^) 96 h, movement and feeding rates.	LC_50_ from 1 for [OMPy][Br] to 580.2 mg L^−1^ for [TBA][Br], Snails moved more slowly when exposed to butyl- and hexyl-cation ILs but were not affected at higher IL concentrations (4–10% of LC_50_) or ILs with octyl alkyl groups. The snails grazed less at higher IL conc.	[119]
[HMIM][Br]	*Daphnia magna*	The lethality rate (%), malformation rates (%).	The IL exposure (21 days) caused a marked drop in the survival, molts, and the number of the first brood. The total number of offspring was declined in the group treated with 1.6 mg L^−1^, and increased in 0.2 mg L^−1^ group. No effect on sex differentiation was found.	[120]
[C_n_MIM][NO_3_] (*n* = 2, 4, 6, 8, 10, 12)	*Chlorella vulgaris, Daphnia magna.*	EC_50_ (mg L^−1^)24, 48, 72, 96 h; the cumulative immobilization data.	The EC_50_ (mg L^−1^)24 h values decreased from 857.8 for [EMIM] to 0.66 for [C_12_MIM].	[121]
[BMIM] with [Cl], [Br], [BF_4_], [PF_6_].	*Daphnia magna*	The number of first-brood neonates, total number of neonates, average brood size; LC_5_ (mg L^−1^) 48 h, LC_50_ (mg L^−1^) 48 h.	LC_50_ ranged from 8.03 to 19.91 mg L^−1^. The 21′days chronic bioassays showed fewer total neonates, first-brood neonates, and average neonates when exposed to lower concentrations (0.3 mg L^−1^) of imidazolium-based ILs than in the presence of Na-based salts at higher concentrations (400 mg L^−1^).	[122]
[IM], [Py], [P], [N] with [PF_6_], [Cl], [CH_3_SO_4_], [EtO_2_PO_2_], [N(SO_2_CF_3_)_2_].	*Selenastrum capricornutum Daphnia magna*	EC_50_ (mg L^−1^) 48 h.	The logIC_50_ values were for *D.magna* in the range from 0.0017 [OMIM][Cl] to 24 [BMIM][PF6]; for S. *capricornutum* in the range from 0.0011 [C_12_MIM][Cl] to 63 [Py][Cl].	[123]
[BMIM][BF_4_], [MOEMIM][(CN)_2_], [MOEMIM][BF_4_].	*Vibrio fischeri, Daphnia magna*	EC_50_ (mg L^−1^) 15 min-*V. fischeri,* EC_50_ (mg L^−1^) 48h-*D. magna*	The EC_50_ (mg L^−1^) 15 min values were in the rang 2.406[MOEMIM][(CN)_2_], -300 [BMIM][BF_4_] for *Vibrio fischeri.*	[124]
Protic (P)-ILs derived from aliphatic amines and organic acids; aprotic (A)-ILs: [IM], [Py] with [Cl].	*Vibrio fischeri, Pseudokirchneriella subcapitata, Lemna minor*	EC_50_ (mg L^−1^)	PILs have EC_50_ values >100 mg L^−1^; AILs are more toxic and show a lower biodegradability potential.	[125]
[PMIM], [BMIM], [HMIM], [OMIM] with [Br]	*Selenastrum capricornutum*	EC_50_ (µM) 48, 72, 96 h.	EC_50_ (24 h) ranged from 1.65 for [OMIM][Br] to 3.46 for [PMIM][Br].	[126]
[C_n_MIM][Br], *n* = 4–16	*Ulva lactuca*	LC_50_ (4 days)	The [C_12_MIM][Br] triggers the generation of reactive oxygen species (ROS viz. O_2_•–, H_2_O_2_, and OH•), damages of the membrane and DNA (>50–70% increase in % tail DNA over control), inhibits antioxidant systems, accumulates of fatty acids, enhances enzyme activity: SOD, APX, GR by 1.3–2.0-fold, GSH-Px decreases with a higher regeneration of AsA, GSH.	[127]

Abbreviations: SOD, superoxide dismutase; APX, ascorbate peroxidase; GR, glutathione reductase; GSH-Px, glutathione peroxidase; AsA, reduced ascorbate; GSH, reduced glutathione.

**Table 3:** Toxicity of ionic liquids against plants and organisms living in the soil.

Ionic Liquids	Plants and Other Organisms Living in the Soil	Kind of Tests	Observations	Ref.
[EMIM], [BMIM}, [OMIM] with [Cl], [BF_4_], [8OSO_3_], [(CF_3_SO_2_)_2_N)], [(CF_3_)_2_N)], [(2-OPhO)2B].	*Triticum aestivum, Lepidium sativum, Folsomia candida*	Growth inhibition, Reproduction inhibition, EC_50_ (µM kg^−1^ dry weight soil)	Growth inhibition wheat: from 3500 for [BMIM][(CF_3_)_2_N] to 110 for [BMIM][(CF_3_SO_2_)_2_N]; Growth inhibition cress: from 3500 for [BMIM][(CF_3_)_2_N] to 400 for [BMIM][(CF_3_SO_2_)_2_N]; Reproduction inhibition springtail: from >4400 for [BMIM][(CF_3_)_2_N] to 30 for [BMIM][(CF_3_SO_2_)_2_N].	[54]
[EMIM][Cl], [BMIM][Cl] [HMIM][Cl]	*Lepidium sativum L.*	Phytotoxicity tests (chlorosis, necrosis, and leaf and stem deformation); relative growth—RG (%) and EC_50_.	EC_50_ values for [EMIM][Cl] is 0.1046 mg ml^−1^; [BMIM][Cl]—0.0510 mg ml^−1^, [HMIM][Cl]—0.0122 mg ml^−1^.	[140]
[EMIM] with [Br], [NO_3_], [Tos], [dMP], [MS]	spring barley common radish	Plant growth inhibition test; Digital photographs of pots with planted seeds: LOEC, NOEC	ILs showed the highest adverse action to the spring barley (NOEC100 mg/kg dry soil mass). The common radish showed high tolerance to [EMIM][Tos]. The greatest toxic effect showed [EMIM][dMP]. The toxicity of ILs to the growth and development of the early developmental stages of plants was slight.	[143]
[OMIM][Br]	wheat (*Triticum aestivum*) seedlings	Pigment assay, Proline content, Lipid peroxidation, Enzyme activity assay, Dry weight of seedlings	After 7 day IL-treatment by 8 mg L^−1^: pigments content was 56.59%, -60.40% lower, dry weight was 47.82% lower, proline in the leaves of wheat seedlings increased by 11-fold of control, oxidative damage or cell membrane disruption, SOD(−20.6%), CAT(−63.8%), POD(−64%), and APX (91.9%) in the leaves decreased.	[144]
[BMIM][BF_4_]	*Triticum aestivum*	Shoot length of wheat seedlings, The activity of amylase, POD; chlorophyll content.	IL at the conc. > 0.9 mM was toxic to wheat seedlings, germination was reduced to 38.0% in the presence of 4.4 mM [BMIM][BF_4_] compared with the control, The activities of amylase, POD increased in shoots and roots, but it decreased when the IL conc. exceeded 1.8 mM.	[145]
[BMIM][BF_4_], [BMIM][PF6], [BMIM][NTf_2_]	Rice, capsicum	GR, GP on the 7th day; The toxicity to the cell membrane; the content of inorganic salts in the cells; reducing sugars, EC_50_ (g L^−1^).	EC_50_ on stems of rice: [TF_2_N] (1.038) > [PF_6_] (2.450) > [BF_4_] (4.986); for capsicum: [BF_4_] (1.438) > [TF_2_N] (2.254) > [PF_6_] (3.465).	[146]
[C_n_MIM][Cl] *n* = 4, 8, 14	*Caenorhabditis elegans*	The lethality, LC_50_ (mg mL^−1^).	When animals were exposed to 1.0 mg mL^−1^ IL, the lethality went from 0.0% with [BMIM] to 11% with [OMIM], to 97% with [C_14_MIM].	[147]
[OMIM][Br]	*Eisenia fetida*	The growth, reproductive ability, and ATPase activity	5 mg kg^−1^ of exposure to IL inhibited the growth, and reproductive ability; 40 mg kg^−1^ inhibited the activities of ATPase after 3 and 7 days of exposure.	[148]
[BMIM]Cl	Female Fischer 344 rats	Dermal, eye irritation tests; LLNA; EC_3_, LD_50_	LD_50_ was 550 mg kg^−1^.	[149]

Abbreviations: LOEC, the lowest observed effect concentration; NOEC, no observed effect concentration; SOD, superoxide dismutase; CAT, catalase; POD, peroxidase; APX, ascorbate peroxidase; LLNA, local Lymth Node assay; GR, the germination rate; GP, the rooting rate.

**Table 4:** Reports on ILs cytotoxicity.

Ionic Liquid	Cell Cultures	Results	Ref.
[C_n_MIM] ILs, *n* = 4, 6, 10 with [BF_4_], [PF_6_], [Cl], [BEIM][BF_4_]	The human tumor cell line HeLa.	EC_50_ (mM) 24, 44 h values are lowest for [BMIM][BF_4_] (0.6, 5.3).	[52]
[IM], [Py], [Chol] with [Br], amino acids [AA], cystine, arginine, glutamine, glutamic acid.	The human cell HeLa (cervical carcinoma).	The 50% viability is reached near 4 × 10^3^ µM for [HMIM][Br] and [C_6_Py] [Br], for [Chol][AA] was above 4 × 10^3^ µM.	[69]
Protic (P) ILs (aliphatic amines) aprotic (A) ILs [IM], [Py].	The promyelocytic leukemia cells from the rat IPC81 cell line.	The EC_50_ values for the analyzed PILs ranged between 1.76 and 30.95 mM, while the AILs had EC_50_ between 0.1 and 5 mM.	[125]
Phosphonium, and ammonium-based ILs with [PF_6_], [BF_4_], [(CF_3_SO_2_)_2_N], [(C_2_F_5_)_3_PF_3_].	The NCI 60 cell lines: melanoma, CNS, colon, Non-small cell lung, leukemia, renal, ovarian, prostate and brain	[P]-based ILs were found to be more active and less cytotoxic as compared to [N] ILs. Antitumor activity (GI_50_ µM) and toxicity (LC_50_ µM) 24 h were the lower for phosphonium-based ILs: [PF_6_] (−7.01, −4.34), [(CF_3_SO_2_)_2_N] (7.04, −5.28), which were the most active.	[168]
[MPPyrr], [MBPyrr] [MPPip], [MBPip], [MOPyrr] [MOPip] with selected anions.	The human breast cancer cell, line MCF7	[MPPyrro][Br] showed the lowest toxicity with 43.4 IC_50_ (mM), [Octyl-methyl-Pyrro] [Br] showed the highest toxicity with 0.078 IC_50_ (mM).	[173]
[C_n_MIM] *n* = 1, 4, 6, 8, 10; with [Cl], [PF_6_], [MSO_4_], [MEIM][ESO4], [BnMIM] [p-FBnMIM] [p-ClBnMIM] with [Cl]	The human Caco-2 cell line.	The most toxic IL was [C_10_MIM] [Cl] with EC_50_ (mM) 24, 48 h 0.03; 0.01 whereas the least toxic was [MMIM][MSO_4_] with 81.24; 31.69 values.	[174]
[MIM], [Py], [Chol], [N], [P] with [Cl], [Br], [BF_4_], [Tf_2_N].	The human cell line HeLa.	The EC_50_ (mM) 48 H values were in the range: for [MIM] 0.19-9.94; [Py] 1.04–2.90; [Chol] 0.98–3.08; [N] 0.68–1.74; [P] 2.24–2.50.	[175]
[CnMIM], *n* = 4, 5, 7, 10 with [BF_4_], [PF_6_], [Br], [Tf_2_N].	the fish CCO cell line	The EC_50_ (mM) 72 h values were in the range <0.1 for [C_7_MIM] [Tf_2_N], [C_10_MIM][Tf_2_N] to >10 for [BMIM][PF_6_].	[176]
[MIM], [DMG], [P], [N], [Chol] with [BF_4_], [PF_6_], [(CN)_2_N], [Tf_2_N], saccharin, acesulfame.	The human colon carcinoma HT29 and CaCo-2 cell lines	[BMIM], [C_2_OHMIM], [C_5_OHMIM], [Chol] are no toxic, [C_8_MIM][BF_4_], [C_10_MIM][BF_4_] showed log EC_50_ (µM)-HT-29 and CaCo-2: (3.6; 3.34), (2.46; 2.78). The most toxic ILs were [DMG][PF_6_], Aliquat acesulfame and Aliquat saccharin.	[177]
[DDA][Sac]	LoVo and DLD-1 (colorectal adenomas), HepG2 (liver cancer), AGS (stomach adenoma), A549 (lungs cancer), HaCaT (immortal human keratinocyte).	IC_50_ values tested on six human cell lines varied from 1.44 µM to 5.47 µM.	[178]
[CDHP], [CDBP], [CBEH], [CTMP], [CDEP]	The J774 murine macrophage cell line	The EC_50_ values (mM) of CDHP (20), CDBP (9.1), and CDEP (8.2) were lower than, [CDEP](8.2), [CTMP](0.25), [CBEH](0.30).	[179]
Amidinium, [IM], [P]-based ILs	The human corneal epithelial cells	IL solutions (2.2 × 10^−4^ to 16.7 mM, 10^−5^ to 10^−1^%, *w*/*v*), i. Amidinium and imidazolium ILs showed no significant effect on the cells, within the concentration range 0.02 mM. [P4444]Cl does induce a significant stress response at higher conc.	[180]
Imidazolium ILs encompassing benzothiazole ring and an amide linkage	The colon cancer cell lines (HCT-116 and Caco-2) and breast cancer cell lines (T47D and MCF-7).	The IC_50_ (µM) 48 h value for the most promising IL was in the range 88–115.	[181]
Fluorinated pyridinium salts-based hydrazones	The tumor cell lines (human ductal breast epithelial tumor T47D, human breast adeno- carcinoma MCF-7, human epithelial carcinoma HeLa, human epithelial colorectal adenocarcinoma Caco-2)	ILs with promising antitumor activity showed LD_50_ values (ng/µL) in the range: 278–301, 498–528, 463–486.	[182]
[IM], with [Br], [Cl], [BF_4_], and [Pip], [Pyr], [N_1111_], [P_4444_] with [Br]	*Spodoptera frugiperda* 9Sf-9) cell lines.	The IC_50_ (mg mL^−1^) 24 h values were in the ranges for [IM], [Pip], [Pyr], [N_1111_], [P_4444_]: 0.0037–1.7070; 4.2580, 0.6813; 12.4400; 0.9915.	[183]

Abbreviations: CNS—central nervous system; GI_50_—50% growth inhibition.

**Table 5:** Examples of ILs with pharmaceutical potentials.

Cation	Anion	Activity	Ref.
Benzalkonium [BA]	Saccharinate [Sac], chloride [Cl], acesulfamate [Ace]	Antimicrobial	[125,207,208,209]
Didecyldimethylammonium [DDA]	Chloride [Cl], acesulfamate [Ace], Saccharinate [Sac]	Antimicrobial, antiseptic	[5,70,166,205,206,209]
Tetrabutylphosphonium, tetractylammonium	trifluorotris(perfluoroethyl) phosphate, bis[(trifluoromethyl) sulfonyl]amide	Anti-cancer	[168]
1-alkylquinolinium, 1-alkyl-3-methylimidazolium, 3-methyl-1-tetradecyl-1H-imidazol-3-ium	Bromide, chloride	Anti-biofilm	[209]
Lidocaine hydrochloride	Sodium docusate	Regional anesthetic/an emollient	[3]
Ranitidine hydrochloride	Sodium docusate	Antiulcer: histamine H2-receptor antagonist/emollient	[3]
Didecyldimethylammonium bromide	Ibuprofenate	Antibacterial/anti-inflammatory	[3]
Didecyldimethlammonium	Penicillin G	Antibiotic	[210,218,219]
Didecyldimethylammonium	Ibuprofenate	Anti-inflammatory/analgesics	[210,218,219]
Hexadecylpyridinium	Valproate	Antiepileptic	[210,218,219]
Phenazone	Gentisic acid	Analgesic, anti-inflammatory, antipyretic	[220]
Benzalkonium:	Ibuprofenate	Antibacterial/anti-inflammatory	[221]
Didecyldimethylammonium	Ibuprofenate	Antibacterial/anti-inflammatory	[3]
Benzalkonium	Colawet MA-80	Antibacterial/wetting agent	[221]
Benzalkonium	Sulfacetamide	Antibacterial/anti-acne	[221]

## Abbreviations

[IM]	imidazolium
[Mor]	morpholinium
[N]	quaternary ammonium
[Pip]	piperidinium
[Py]	pyridinium
[Pyr]	pyrrolidinium
[MMIM]	1-methyl-3-methyl imidazolium
[EMIM]	1-ethyl-3-methyl imidazolium
[PMIM]	1-propyl-3-methyl imidazolium
[BMIM]	1-butyl-3-methyl imidazolium
[HMIM]	1-hexyl-3-methyl imidazolium
[OMIM]	1-octyl-3-methyl imidazolium
[DMIM]	1-decyl-3-methyl imidazolium
[BzMIM]	1-benzyl-3-methyl imidazolium
[BnMIM]	1-benzyl-3-methyl imidazolium
[p-FBnMIM]	1-p-fluorobenzyl-3-methyl imidazolium
[BnEIM]	1-benzyl-3-ethyl imidazolium;
[p-ClBnMIM]	1-p-chlorobenzyl-3-methyl imidazolium
[TMSiMMIM]	1-methyl-3-[(trimethylsilyl)methyl]-imidazolium;
[C_2_OHMIM]	3-(2-hydroxyethyl)-1-methyl imidazolium
[C_2_ClMIM]	3-(2-cloroethyl)-1-methyl imidazolium
[C_2_ClMIM]	3-(2-cloroethyl)-1-methyl imidazolium
[C_3_OHMIM]	3-(2-cloropropyl)-1-methyl imidazolium
[MOEMIM]	1-methoxyethyl-3-methyl imidazolium
[C_10_C(O)OEMIM]	3-methyl-1 (ethoxycarbonyloctyl)imidazolium
[BMPyr]	1-butyl-3-methyl pyridinium
[HMPyr]	1-hexyl-3- methyl pyridinium
[OMPyr]	1-octyl-3-methyl pyridinium
[Py-4NMe2′]	dimethylamino pyridinium
[BMPy]	1-butyl-3-methyl pyridinium
[HMPy]	1-hexyl-3-methyl pyridinium
[OMPy]	1-octyl-3-methyl pyridinium
[BMPyrr]	1-butyl-1-methyl pyrrolidinium
[MPPyrr]	1-methyl-1-propyl pyrrolidinium;
[MBPyrr]	1-methyl-1-butyl pyrrolidinium
[MOPyrr]	1-methyl-1-octyl pyrrolidinium
[MPPip]	1-methyl-1-propyl piperidinium
[MBPip]	1-methyl-1-butyl piperidinium
[MOPip]	1-methyl-1-octyl piperidinium
[P_6,6,6,14_]	trihexyltetradecylphosphonium
[TBP]	tetrabutylphosphonium
[P_4,4,4,4_]	tetrabutylphosphonium
[P_4,4,4,1_]	tributyl (methyl)phosphonium
[N_1,8,8,8_]	methyltrioctylammonium
[TBA]	tetrabutylammonium;
[DDA]	didecyldimethylammonium
[TBI]	2-mercaptobenzimidazole
[TBO]	2-mercaptobenzoxazole
[TBP]	tri-*n*-butyl phosphate
[TBT]	2-mercaptobenzothiazole
[TMGC4]	di-butyltetramethyl-guanidinium
[TMGC7]	di-heptyltetramethylguanidinium;
[TMGC12]	di-dodecyltetramethylguanidinium
[(di-h)2DMG]	tetrahexyldimethylguanidinium
[(C_3_O)_4_DMG]	N”N”-dimethyl-N,N,N’,N’-tetra-(2-methoxyethyl)-guanidinium
[EMMor]	ethyl-methylmorpholinium
[EBMor]	butylethylmorholinium
[Chol]	choline
[Br]	bromide
[Cl]	chloride
[I]	iodide
[PF_6_]	hexafluorophosphate
[NTf_2_]	bis(trifluoromethylsulfonyl)imide
[BF4]	tetrafluorobotare
[Tos]	tosylate;
[Sac]	saccharine
[C_8_H_17_SO_4_]	octylsulfate
[CF_3_SO_3_]	trifluoromethanesulfonate
[SbF_6_]	hexafluoroantimonate
[ESO_4_]	ethyl sulfate;
[C_2_C_2_C_2_S]	triethylsulfonium
[ETHT]	ethyltetrahydrothiophenium
[8OSO_3_]	octylsulfate
[(CF_3_SO_2_)_2_N]	bis(trifluoromethylsulfonyl)imide
[(CF_3_)_2_N]	bis(trifluoromethyl)imide
[(2-OPhO)_2_B]	bis(1,2-benzenediolato)borate
[CH_3_SO_4_]	methylsulfate
[CH_3_SO_3_]	methanosulfonate
[dMP]	dimethylphosphate
[MS]	methanesulfonate,
[CDHP]	dihydrogen phosphate
[CDBP]	dibutyl phosphate
[CBEH]	bis(2-ethylhexyl) phosphate
[CTMP]	bis(2,4,4-trimethylpentyl)phosphinate
[CDEP]	*O,O’*-diethyl dithiophosphate
